# Geometric Morphometric Versus Genomic Patterns in a Large Polyploid Plant Species Complex

**DOI:** 10.3390/biology12030418

**Published:** 2023-03-09

**Authors:** Ladislav Hodač, Kevin Karbstein, Salvatore Tomasello, Jana Wäldchen, John Paul Bradican, Elvira Hörandl

**Affiliations:** 1 Department of Systematics, Biodiversity and Evolution of Plants (with Herbarium), Albrecht-von-Haller Institute for Plant Sciences, University of Göttingen, 37073 Göttingen, Germany; lhodac@uni-goettingen.de (L.H.); kevin.karbstein@uni-goettingen.de (K.K.); salvatore.tomasello@uni-goettingen.de (S.T.); johnpaul.bradican@uni-goettingen.de (J.P.B.); 2 Department of Biogeochemical Integration, Max Planck Institute for Biogeochemistry, 07745 Jena, Germany; jwald@bgc-jena.mpg.de

**Keywords:** apomixis, genomics, geometric morphometrics, polyploidy, *Ranunculus auricomus*, taxonomically complex groups (TCGs)

## Abstract

**Simple Summary:**

Plant species complexes with hybridization and asexual reproduction often exhibit complex morphological patterns, which is problematic for classifications. Here, we analyze geometric morphometric, genomic, and ecological data with comprehensive statistics to evaluate phenotypic variation in the Eurasian *Ranunculus auricomus* complex. Genomic clusters correspond largely to morphological groupings, but most described asexual hybrid taxa cannot be discriminated from each other. Phenotypic variation is more influenced by genomic composition than by climatic conditions, and the phenotypic variation of asexual hybrids resembles a mosaic of intermediate and transgressive phenotypes. Our results support a taxonomic revision of the complex.

**Abstract:**

Plant species complexes represent a particularly interesting example of taxonomically complex groups (TCGs), linking hybridization, apomixis, and polyploidy with complex morphological patterns. In such TCGs, mosaic-like character combinations and conflicts of morphological data with molecular phylogenies present a major problem for species classification. Here, we used the large polyploid apomictic European *Ranunculus auricomus* complex to study relationships among five diploid sexual progenitor species and 75 polyploid apomictic derivate taxa, based on geometric morphometrics using 11,690 landmarked objects (basal and stem leaves, receptacles), genomic data (97,312 RAD-Seq loci, 48 phased target enrichment genes, 71 plastid regions) from 220 populations. We showed that (1) observed genomic clusters correspond to morphological groupings based on basal leaves and concatenated traits, and morphological groups were best resolved with RAD-Seq data; (2) described apomictic taxa usually overlap within trait morphospace except for those taxa at the space edges; (3) apomictic phenotypes are highly influenced by parental subgenome composition and to a lesser extent by climatic factors; and (4) allopolyploid apomictic taxa, compared to their sexual progenitor, resemble a mosaic of ecological and morphological intermediate to transgressive biotypes. The joint evaluation of phylogenomic, phenotypic, reproductive, and ecological data supports a revision of purely descriptive, subjective traditional morphological classifications.

## 1. Introduction

Polyploidy and hybridization are regarded as key factors for plant evolution [1,2,3,4,5]. Polyploidy, the presence of more than two chromosome sets within a cell, has several positive evolutionary consequences. Multiple gene copies allow for higher gene expression along with higher physiological (and thus phenotypic) flexibility in relation to abiotic and biotic environmental conditions [6,7]. Polyploids thus often perform better in past glaciated areas, under climatic change, or in the colonization of new ecosystems [8,9,10]. In addition, hybridization, the fusion of previously diverged subgenomes, leads to new genetic combinations, increased heterozygosity, hybrid vigor, buffering of deleterious mutations, and changes in secondary metabolites [11,12]. Nevertheless, newly formed polyploids may have reduced fertility due to meiotic errors [13,14,15], but they can escape hybrid sterility via asexual reproduction and/or selfing [5,9,13,16]. Apomixis, the asexual reproduction via seeds, occurs in c. 19% of families and c. 2% of genera in flowering plants [17,18]. Hybridization is probably the main trigger of apomixis [16,19,20]. Apomixis is heritable and genetically controlled but usually facultative because it represents a modification of the sexual pathway [20,21,22]. The extant positive side-effects of polyploidy and hybridization are ‘fixed’ over generations and can foster the establishment of apomictic lineages in new or stressful environments (e.g., in previously glaciated areas; [9,23,24]).

Taxonomy, i.e., documenting, classifying, naming, and understanding the diversity of life, represents a cornerstone of biological research [25,26,27]. More than two million eukaryotic species have been described thus far, but many species remain undiscovered or unnamed [28,29,30]. Species are the fundamental units of evolutionary and biodiversity research (e.g., ecology or nature conservation). Traditional plant taxonomy has a long historical background and was based until the 1970s almost exclusively on morphological distinctness (reviewed by [31]). The subjectivity of defining “distinctness” by descriptive methods, and the recognition of different evolutionary processes leading to distinct entities have led to many different species concepts and pluralistic views [31]. Phylogenetic lineage concepts can be further problematic in cases of reticulated evolution [1]. For hybridizing complexes with few intermediates, cluster species concepts based on phenetic or genetic similarity have been proposed [32]. To better recognize evolutionary processes, modern authors consider species as separate genetic ancestor-descendent lineages, a concept that applies to diploids, polyploids, sexuals, and asexuals [33,34,35,36]. Criteria from previous concepts should now be applied to analyze and describe the evolutionary role and circumscription of lineages, e.g., their persistence in time and space or phenotypic differentiation, which is still an obstacle [1,33,37,38,39,40]. The current era of genomics has enabled astonishing breakthroughs in high-throughput sequencing (HTS) of DNA, computation capabilities, and bioinformatics, resulting in a plethora of new evolutionary insights and subsequent taxonomic revisions and species descriptions in the plant kingdom [4,36,39,41]. Despite all this progress, awareness is increasing that not all lineages necessarily represent species. The currently most accurate model for species delimitation (“Multispecies Coalescent”, or MSC) tends to oversplit groups into many species [35,39,42]. For example, information on geographical isolation can provide insight into whether observed lineages represent populations or species [39]. Additional criteria are therefore needed for the formal classification of lineages. Recognition of genetic and/or morphological clusters is another timely approach for species delimitation and can be applied to phenotypic and genetic data regardless of the mode of reproduction and the presence/absence of crossing barriers [32,43]. Consequently, an integrative taxon-omics approach that combines taxonomy with 21st-century ‘-omics’ (HTS) and other data sources (e.g., morphology, reproduction, or ecology) excludes discipline-dependent failure rates and is thus considered to be the gold standard in species delimitation [44,45,46,47].

Taxonomically complex groups (TCGs) [48] offer a unique opportunity to study flowering plant evolution. TCGs are groups of related individuals that are characterized by various biological factors that complicate the delimitation of species [48,49]. Apomictic polyploid complexes fit the definition of TCGs; they link intricate microevolutionary processes such as polyploidization, hybridization, and asexuality with macroevolutionary patterns [3,45,50]. Sexually diploid parents usually generate hundreds of hybrid, polyploid hybrid, and/or apomictic derivatives multiple times throughout time and space [38,51,52,53,54]. Particularly, the combination of polyploidy and hybridization (allopolyploidy) frequently shows higher degrees of (epi)genomic and transcriptomic changes than polyploidy alone (autopolyploidy) [3,7,55,56,57] and is thus more likely to create biotypes with novel phenotypic features [58,59,60]. In nature, many distinct autopolyploid cytotypes remain unnamed and hence unrecognized due to only minor morphological differences compared to diploid progenitors [57,61,62]. Concerning apomictic polyploid complexes, [38] reviewed four alternative approaches for a case-by-case classification: (i) classify the obligate sexual progenitors as species; (ii) merge them and highly facultative apomictic lineages into a single species; (iii) treat the main hybrid clusters of the facultative apomicts as species. If the parentage of allopolyploid apomicts can be reconstructed, then designating apomicts as nothotaxa [63] can be a useful approach to formally separate from sexual species [51,64,65,66]; and (iv), classify obligate apomictic lineages as agamospecies. While options (i) and (ii) have been applied in several genera (reviewed by [38]), the challenge remains for (iii) polyploid complexes comprising hundreds and thousands of described taxa with uncertain taxonomic circumscriptions. Only a few case studies using integrative taxon-omics and a combination of ancestor-descendant lineage and cluster criteria have been published thus far (e.g., [39,54,66]). Classification is highly dependent on the degree of apomixis and the stability of lineages in polyploid complexes. For instance, [51] made substantial progress in untying highly reticulate relationships and genome evolution in facultative to obligate apomictic polyploid complexes, but recognizing distinct lineages and their morphotype was nearly impossible due to innumerous reticulations producing large network-like clusters. Another issue for phylogenomic, as well as phenotypic, reconstructions arises when a sexual progenitor is not sampled or presumed to be extinct, and consequently, its morphotype remains unknown [51,52,67]. For instance, sexual progenitors for some agamospecies are completely unknown (e.g., *Alchemilla*, [68]).

In the last years, many researchers working in the field of integrative taxon-omics focused on bringing their plant model systems into the era of genomics, utilizing either (sub)genomic datasets (e.g., restriction-site associated DNA sequencing (RAD-Seq) or target enrichment of nuclear genes (TEG)) and/or a combination of the different genomic, nuclear gene, and plastid regions (e.g., *Cardamine*, *Leucanthemum*, *Ranunculus auricomus*, *Rubus*, or *Salix*) [36,51,69,70,71,72,73,74]. Gathering information to infer lineage characteristics and subsequent species delimitation, e.g., shared or distinct morphotypes, is still regarded as an important criterion for species delimitation [31,36,37,51]. However, this is often done using traditional morphological descriptions, morphometrics, or character evolution approaches to modeling character state changes within a phylogeny [31,75]. In the past, purely descriptive traditional morphological classification led to subjective descriptions of hundreds to thousands of morphotypes as species due to minor morphological differences in TCGs (e.g., [76,77]), a practice that was particularly prevalent in apomictic polyploid complexes (e.g., [50,78,79]). Delimitation that only relies on single, partly author-dependent ‘diagnostic’ characters bears the danger of subjective, irreproducible taxonomic classifications. Therefore, analysis using multiple characters is preferred to more objectively characterize different phenotypes [31,39,80]. Another challenge for species delimitation is the exclusion of non-relevant variation of characters (e.g., allometry or asymmetrical development of organs), which is a relevant factor in plants due to large phenotypic plasticity in response to environmental factors [31,81,82,83,84].

Geometric morphometrics (GM, or GMM by recent publications; e.g., [85,86]) tackles the aforementioned issues through the exact, objective, and fine-scale evaluation of shapes and shape changes via landmarks (i.e., anatomical loci) [87,88,89,90]. This approach has been applied across many disciplines (e.g., botany, paleontology, medicine, or engineering), and uses a collection of multivariate statistical analysis to visualize Cartesian coordinate data [83,91]. In plant research, leaf shapes were frequently analyzed for species characterization and delimitation [88,92]. However, GM approaches can also be easily extended to other structures possessing shared biologically homologous regions in a specific study group, e.g., receptacle shape in [39], or 3D flower shape in [93,94]. In general, morphological shape changes are associated with (epi)genetic variation and environmentally related responses [88,95,96,97,98]. Nevertheless, to the best of our knowledge, no study so far has inferred interspecific, landmark-based, multi-trait shape changes across large geographic scales for species delimitation. Moreover, GM is also able to support final taxonomic decisions based on HTS data, particularly in TCGs where morphological differences are hard to assess with traditional morphological approaches [39,99,100]. Combinations of phylogenetic and -omic data with landmark-based GM approaches are effective in disentangling intricate plant species relationships [39,101]. However, in plant species complexes, the most challenging aspect is the aforementioned delimitation of young allopolyploid derivatives. In TCGs with porous genomes (i.e., some genomic regions are protected from interspecific gene flow, whereas others are not) and hybridization, mosaic-like character combinations and conflicts of morphological data with molecular phylogenies are a major problem for classification (reviewed, e.g., by [38,101,102]). Consequently, it remains to be tested whether these landmark-based GM approaches using multi-trait data are suitable for resolving highly reticulate TCGs.

Concerning species delimitation in TCGs, different phylogenomic approaches are available to efficiently resolve intricate relationships. RAD-Seq collects non-coding and coding regions across the entire genome and delivers thousands to hundreds of thousands of loci and SNPs [103,104]. This provides a particularly powerful method for tackling TCGs characterized by low genetic divergence, reticulations, and ILS [51,69,71]. However, RAD-Seq loci are usually short and insufficiently informative to allow for reliable allele phasing. Retrieving allelic information and discriminating homoeologous loci is particularly crucial for accurate inferences of reticulate polyploid relationships [67,105,106,107]. Single-copy nuclear genes assembled from TEG are often longer than RAD-Seq loci, enabling the segregation of alleles at a single locus (i.e., phasing), and thus MSC approaches [104,108,109]. Therefore, phylogenomic analyses conducted with TEG datasets can more clearly delimit the genetic structure of polyploid complexes, differentiate between allo- and autopolyploid evolution, and determine the parentage of a single polyploid [51,73,107]. In addition, data from plastid regions or entire plastomes (CP) can be easily gained from TEG off-target reads [110,111]. Together with RAD-Seq and TEG, these help to identify reticulations (nuclear-plastid discordances), homoploid speciation, extinction of sexual progenitors, allopolyploidization events, and/or maternal progenitors of polyploids [51,73,112,113]. Despite all this progress, detailed morphological characterizations of all those lineages/clusters found by modern phylogenomic approaches are often missing in these studies. Consequently, there is a need to inform these lineages/clusters and their evolutionary reconstructions by using detailed morphological characteristics obtained from comprehensive landmark-based, multi-trait GM datasets informed by subgenomic data. Additionally, knowledge is missing on which genomic dataset (RAD-Seq, TEG, or CP) best fits observed morphological differentiation.

**Figure 1:**
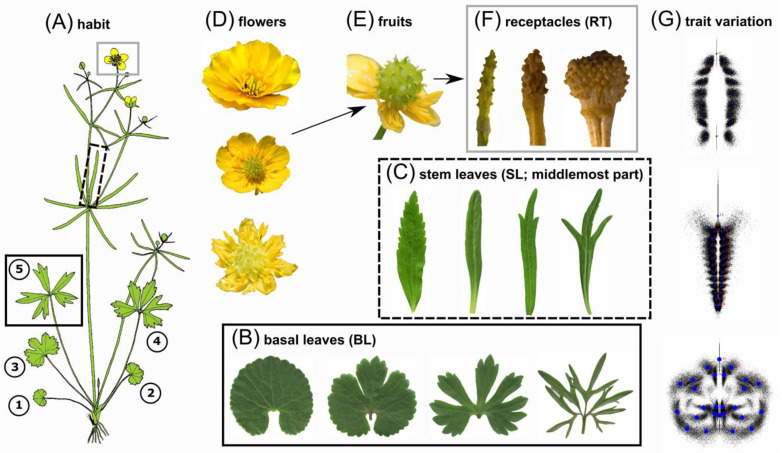
Morphological variation of taxonomically informative traits within the *R. auricomus* species complex (see also Figure S1A–C for leaf venation and landmark configurations per trait). (**A**) Illustration of a typical *R. auricomus* individual with morphological traits highlighted in boxes: basal leaf cycle (black box; 1–5, 1–4 = early spring leaves, 5 = most dissected leaf at anthesis) and stem leaves with the middlemost segment (black-dotted box) and reproductive structures (grey box; flower, fruit, and receptacle at the fruiting stage). Figure source: The figure was taken from [50], which is published under the Creative Commons Attribution License (**B**) Variability of basal leaves among taxa ranging from undivided (left) to broad three-lobed or dissected (center) to strongly dissected forms (right). (**C**) Variability of the middlemost segment of a stem leaf among taxa, ranging from broadly lanceolate (with teeth; left) to narrowly linear forms (with dissection; right). (**D**) Variability of petal variation, ranging from five to 15 (mostly sexuals; the uppermost flower) or reduced or absent forms (mostly polyploid apomicts; the lowermost flower). (**E**) Collective fruit in the ripening process: Concerning polyploid apomicts, a single collective fruit can contain achenes with either sexually or apomictically produced seeds. (**F**) Variability of receptacles among taxa, ranging from narrowly large (left) to smaller roundish receptacles (right). (**G**) The variation of the evaluated traits captured by GM is shown as clouds of 2D landmarks. Photographs of Figure 1B–F: © Kevin Karbstein.

The *Ranunculus auricomus* plant complex is a model system for apomixis research but also for studying the evolution of phylogenetically young TCGs [13,21,51,64,87,88,114,115]. Within the genus *Ranunculus*, the group falls into a large clade, with its closest relatives occurring in North America and Central Asia [116,117]. The distribution of taxa ranges from Greenland to Europe, Northern Asia, and Alaska; it spans arctic, boreal, temperate, and Mediterranean climate zones [118,119,120]. Taxa occupy various habitats—from stream- and riverside habitats, alluvial to humid deciduous forests, extensively used swampy to semi-dry meadows, and waysides [50,121,122,123]. The complex comprises more than 800 taxa [124,125] that were predominantly described by applying descriptive morphological species concepts (e.g., [122,126,127,128,129]). The existence of two remarkably different morphotypes already led Linnaeus in 1753 [130] to classify the complex into two different species: *R. auricomus* L. from Western Europe, characterized by dissected basal leaves, and *R. cassubicus* L. from North Poland or further east (Siberia), with large non-dissected basal leaves [130,131] (Figure 1A,B). In the 19th century, intermediate morphotypes between these two taxa occurring in Central Europe, Sweden, and Finland were described as *R. fallax* (Wimm. & Grabowski) Sloboda [132], and in 1922 some dwarf arctic-alpine morphotypes from Siberia were discriminated as *R. monophyllus* Ovcz. [133]. These four morphotypes established a widely used classification of four main species with several subspecies [122,134], which was used in many European floras. Subsequently, hundreds of different, partly only locally occurring, morphospecies have been described, connecting these four core groups by endless intermediates (e.g., [122,126,127,135,136,137,138]). However, these morphology-based species concepts failed either due to an inability to split the morphotype continuum or the presence of intricate evolutionary processes [122,134,139,140]. Consequently, the complex is often treated as an agglomerate in regional floras (e.g., [141]), neglecting its biodiversity.

**Figure 2:**
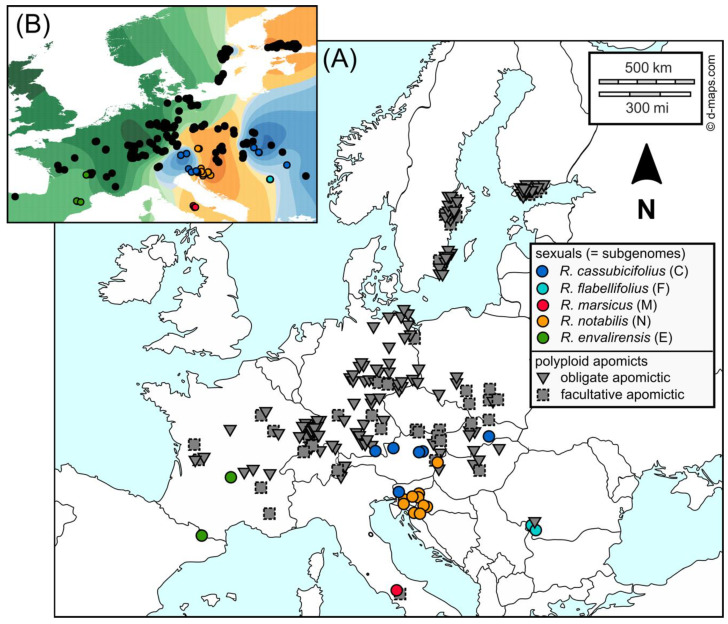
Sampling localities of studied *Ranunculus auricomus* populations across Europe (details in Table S1). (**A**) Symbols represent the reproduction modes of populations (colored circles = diploid to tetraploid sexual; dark gray solid triangles = polyploid obligate apomicts; dark gray dashed squares = polyploid facultative apomicts; [24]). The color scheme was also applied to Figures 3–7, and 9. The original map was downloaded from https://d-maps.com/ (accessed on 8 October 2020), created by [24,51] which are published under the Creative Commons Attribution License, and modified herein. (**B**) Geographic map illustrating the ancestry coefficients of the likeliest genomic resolution (K = 3 RAD-Seq clusters) across Europe according to sNMF analyses published in [51]. These genetic clusters are comparable to RADpainter clusters, which are used here in statistical analyses (Figures 3–7 and 9). Black circles indicate apomictic polyploids, whereas colored circles represent sexual species (similar population sampling as in [51]), and geographic regions (with polyploids) are colored according to the dominant genomic contribution from the respective sexual progenitor species. The figure was created by [51] (published under the Creative Commons Attribution License) and modified herein.

The *R. auricomus* complex is composed of a few, mainly diploid sexual progenitors and hundreds (>800) of polyploid apomictic derivatives. Sexual species are characterized by complete flowers, whereas obligate facultative apomicts exhibit rather reduced flowers with fewer or no petals (the petaloid nectary scales of *Ranunculus* are here conveniently called ‘petals’) [24] (Figure 1D). Taxa have a heterophyllous basal leaf cycle, i.e., usually starting with a non-dissected to three-lobed spring leaf or a basal sheath. The subsequent leaves are more and more dissected and appear during anthesis, but such dissected leaves can also be missing under unfavorable environmental conditions; non-dissected to three-lobed leaves appear during the fruiting stage and persist over summer and autumn (Figure 1A,B; development and homology of three different types of leaf cycles are explained by [120,121,142]). Phylogenomic analyses based on subgenomic data (RAD-Seq, TEG) and GM revealed five geographically isolated, genetically distinct sexual progenitors [39] (Figure 2A,B). Speciation took place ca. 830,000–580,000 years ago and was triggered by vicariance processes during a time frame of severe climatic fluctuations [143]. Based on previous studies [88,121,144], [39] developed a landmarking scheme for the taxonomically most informative traits: (i) the most-dissected basal leaves in the leaf cycle during anthesis; (ii) the central part of the lowermost stem leaf; and (iii) receptacle at fruit stage (Figure 1A–F, Figure S1). The diploid and partly autotetraploid species *R. cassubicifolius* and the diploid, probably homoploid hybrid species *R. flabellifolius* are distributed in Central and Eastern Europe and are characterized by a leaf cycle without dissected basal leaves, but during anthesis, the non-dissected to three-lobed ‘summer’ leaves are already present; *R. cassubicifolius* has broad lanceolate stem leaf segments, whereas *R. flabellifolius* forms a fan-shaped stem leaf with connate segments [51,115,127,128,145,146] (Figure 1B,C; Figures 6 and S8 in [39]). The other sexual species are characterized by a heterophyllous leaf cycle with dissected basal leaves at anthesis. The diploid *R. envalirensis* and the only exclusively tetraploid (probably allotetraploid) sexual *R. marsicus* inhabit restricted ranges in the Southern European mountain systems. These dwarf species show basal leaves with three- to five-lobed or dissected segments and linear stem leaf segments (with sinuses in the case of *R. marsicus*; [39,51,128,147,148]). In contrast, *R. notabilis* is widely distributed in the Illyrian lowlands, is taller, and has rather narrowly lobed or dissected basal leaves and mostly linear stem leaf segments [39,128,137].

Further comprehensive phylogenetic and phylogenomic studies demonstrated that the evolutionary history of the *R. auricomus* complex is substantially shaped by hybridization among sexual progenitors combined with polyploidization [51,64,87,88,115]. Recently, an integrative approach based on subgenomic data (RAD-Seq, TEG, CP), ploidy, and reproductive data with appropriate polyploid bioinformatic tools revealed (i) that only five diploid sexual progenitor species (including an unknown progenitor) probably generated a large number of diverse polyploid apomicts; three to five allopolyploid genetic clusters including progenitor species were characterized by substantial post-origin genome evolution and subgenome dominance [51]. However, it is unclear whether these clusters can also be morphologically recognized. The study revealed further that almost all previously described morphospecies were polyphyletic and did not represent stable ancestor-descendant lineages. The question remains whether these hybrid biotypes (provisorily treated as nothotaxa) would exhibit specific phenotypic variation that would be more extreme or new, or the ability to settle new abiotic and biotic environments compared to their progenitor species. Such morphotypes could eventually result from transgressive segregation and hybrid speciation [149,150]. Transgressive segregation might have occurred in the initial, mostly sexually formed *R. auricomus* hybrid generations [16].

Consequently, we aim at addressing the following questions in this study: (1) Do the genetic clusters found by [51] correspond to morphological clusters? Which genomic dataset (genomic, nuclear, or plastid) is most congruent with the morphological clustering? (2) Is the GM approach of [39] able to delineate the polyploid apomicts from each other and the sexual species? Which are the most informative traits? Do any of the described nothotaxa form well-differentiated morphological clusters? (3) Are morphological shape changes associated with environmental factors or, rather, with genetic factors? (4) Are polyploid apomicts inside or outside the morphospace or ecological niche of the diploid sexual progenitors? We will focus here on the huge diversity of temperate to submeridional taxa that were genetically analyzed by [51], whereas arctic-alpine dwarf forms (‘*R. monophyllus*’) but also Mediterranean taxa of the complex will be the subject of upcoming studies.

## 2. Materials and Methods

### 2.1. Study Locations and Material Sampling

In the present study, we included 28 populations of all four diploids and one tetraploid sexual species (see taxonomic treatment in [39]) and 192 populations of the ca. 75 most widespread tetra-, penta-, and hexaploid apomictic *R. auricomus* taxa (flow cytometric ploidy and reproduction mode measurements published in [24] and deposited in FigShare https://doi.org/10.6084/m9.figshare.13352429 (accessed on 7 March 2023). A sampling of garden plants took place from 2013 to 2018, totaling 220 populations across temperate and submeridional Europe (Figure 1, Table S1). Per population, we recorded altitude, GPS coordinates, and habitat, and collected herbarium specimens. Details about locations, ploidy, reproduction modes, samples per population, and further genomic and environmental characteristics are given in Table S1. Subpopulations from the same locality were treated as separate populations in subsequent statistical analyses because they are characterized by different taxa (morphotypes). Sampled living plants were kept in the Old Botanical Garden at the University of Göttingen under controlled environmental conditions (garden beds with similar solar radiation and water supply) for GM analyses. Individuals were cultivated in 1.5 l pots with Fruhstorfer Topferde LD 80. Voucher specimens were deposited in the herbarium of the University of Göttingen (GOET).

### 2.2. Genomic and Environmental Data Analysis

Wet lab work, data filtering, assembly, parameter optimization, and bioinformatic data evaluation concerning RAD-Seq, TEG, and CP data are described in detail in [24,39,51]. Demultiplexed RAD-Seq and TEG raw reads are deposited in the Sequence Read Archive (SRA) of NCBI (https://www.ncbi.nlm.nih.gov/bioproject/627796 (accessed on 7 March 2023); https://www.ncbi.nlm.nih.gov/bioproject/628081 (accessed on 7 March 2023)). To clarify which genomic dataset (genomic, nuclear, or plastid) best explains the morphological clustering, we used the (phylo)genomic results of [51] that comprise the same sexual and apomictic populations investigated herein. Consequently, we grouped the GM dataset (see the section below) according to the found clades/clusters in [51]. The following naming of clades/clusters corresponds to the respective sexual progenitor found in each clade/cluster.

The RAD-Seq datasets were applied to a genetic structure (sNMF, [151,152]; 1 SNP/locus (unlinked SNPs), 33,165 loci, 33,165 SNPs, and 55% missing data) and genetic similarity (RADpainter+fineRADstructure, [153]; 97,312 loci, 438,775 SNPs, and 74% missing data) analysis. The sNMF analysis is based on an unlinked single nucleotide polymorphism (SNP) alignment (33,165 loci, 194,083 SNPs, 55% missing data). Ancestry coefficients were calculated with method ‘max’, i.e., at each point, the cluster for which the ancestry coefficient was maximal. The sNMF analysis showed three clusters, i.e., a Western European cluster containing the sexual diploid progenitor *R. envalirensis* (E) and related polyploid apomicts, a Central-Eastern European cluster containing the sexual diploid progenitors *R. notabilis* and *R. flabellifolius* (and tetraploid *R. marsicus*, N + F+M), and related polyploid apomicts, and an Eastern European cluster containing the sexual diploid progenitor *R. cassubicifolius* (C) and related polyploid apomicts, as the likeliest genetic resolution. RADpainter also inferred the same three genetic clusters, although a few incongruences were observed (e.g., between clusters E and N + F+M). The TEG dataset was utilized in a STACEY species delimitation analysis [154], using the most informative, nonhomoplasious, and free-from-paralog-sequences 48 nuclear genes, including allele phasing across all ploidy levels as described in [51]. The STACEY analyses inferred five genetic clusters, i.e., clusters each containing *R. cassubicifolius* (C), *R. flabellifolius* (F), *R. marsicus* (M), *R. notabilis* (N), and *R. envalirensis* (E) with related polyploid apomicts. These results, in contrast to RAD-Seq, better delimit progenitor species and their related polyploid apomicts. The CP dataset was used for a maximum likelihood (ML) tree analysis (RAxML_NG, [155]) based on 71 plastid regions (representing ca. 50% of the expected plastome length), containing at least 50% of samples per region, as described in [51]. The ML tree of plastid data analysis exhibited four genetic clades/haplotype groups, i.e., a clade containing *R. cassubicifolius* and *R. flabellifolius* (C + F) and related polyploid apomicts; a clade only with *R. envalirensis*-related polyploid apomicts (including an unknown and probably extinct *R. envalirensis*-related Central European progenitor U); a clade containing *R. envalirensis* (E), and a clade containing *R. notabilis* and *R. marsicus* (N + M). These results thus substantially differ from RAD-Seq and TEG results and suggest a reticulate evolution of the diploid progenitor *R. flabellifolius* (F) from *R. cassubicifolius* (C) as one putative parent and of the tetraploid *R. marsicus* from at least *R. notabilis* (N), respectively. The ML tree analysis exhibited four genetic clades/haplotype groups, i.e., a clade containing *R. cassubicifolius* and *R. flabellifolius* (C + F) and related polyploid apomicts; a clade only with *R. envalirensis*-related polyploid apomicts (including an unknown and probably extinct *R. envalirensis*-related Central European progenitor U); a clade containing *R. envalirensis* (E); and a clade containing *R. notabilis* and *R. marsicus* (N + M). These results thus substantially differ from RAD-Seq and TEG results and suggest a reticulate evolution of the diploid progenitor *R. flabellifolius* (F) from *R. cassubicifolius* (C) as one putative parent, and of tetraploid *R. marsicus* from at least *R. notabilis* (N), respectively.

The gathering of environmental data from both in situ records and WorldClim databases version 2 [156] including data standardization, is described in detail in [24]. All populations of the concatenated GM dataset are characterized by the following abiotic environmental factors: GPS coordinates (longitude and latitude), altitude (meters above sea level, m. a.s.l.), bioclimatic variables 1–19 in 2.5 min resolution (temperature, precipitation, and their respective seasonality variables), and solar radiation in 2.5 min resolution (kJ m^−2^ day^−1^). We removed autocorrelated variables (*r* > 0.8, Figures S2–S6) from the modeling procedure [157], using the R-package ‘corrplot’ version 0.92 [158] and R version 4.2.0 [159].

### 2.3. Geometric Morphometric (GM) Data Analysis

#### 2.3.1. Data Collection and Preparation

The GM dataset is composed of fresh material sampled from living garden plants and material from herbarium specimens of the same populations (Table S1). We added specimens from different herbaria to supplement the datasets with type material (Table S1, see also [39]). Following the approach of [39] (but see also [88,121,144]), we collected the taxonomically most informative traits of *R. auricomus* individuals, i.e., basal and stem leaves during anthesis and receptacles during the fruiting stage (Figure 1B,C,F). Collections were regularly checked against type specimens to ensure accurate selection. We only used individuals that are characterized by basal leaf, stem leaf, and receptacle traits. As a rule, and as far as possible, several basal leaves, stem leaves, and receptacles were recorded per plant individual, and eight plant individuals were recorded for each population on average. From April to May 2018 and 2019, we harvested, on average, three fresh basal and stem leaves per flowering plant. Leaves were scanned immediately after sampling in 400 dpi resolution using CanoScan LiDE 220 (Canon, Ota, Japan) and Epson Perfection V500 Photo (Seiko Epson, Suwa, Japan) scanners. To increase the statistical robustness but also the accuracy of GM analysis, we additionally digitized the taxonomically most informative leaf traits of selected herbarium specimens with the Herbscan Light Box (including a digital camera with 50.6 megapixels) of the GOET herbarium. Herbarized plant material might exhibit allometric shape changes during the drying process [83,160]. However, the number of analyzed herbarium scans was relatively small compared to the garden material in our dataset. Because of the careful selection of non-type and type herbarium material, which also captures in situ specific phenotypic plasticity, the inclusion of these data makes the statistical analyses more robust and accurate. Moreover, we collected three receptacles per individual on average at the fruiting stage from June to August 2018 and 2019. Receptacles were digitized with 10–15-fold magnification under a Leica M125 microscope (Leica Microsystems, Wetzlar, Germany).

In total, the concatenated dataset comprised 4070 basal leaves, 4148 stem leaves, and 3472 receptacles based on 1858, 1880, and 1587 individuals, respectively (images and landmark files are stored in Figshare https://doi.org/10.6084/m9.figshare.21393375) (accessed on 7 March 2023). Information for five sexual taxa, 64 apomictic polyploid taxa with taxonomic assignment, and another 17 apomictic polyploid taxa without taxonomic assignment (‘cf’, or ‘indet’) were recorded. The total dataset comprises 2048 individuals from 220 populations. The majority (73%) of digitized plant material was derived from garden cultures (University of Göttingen) and was supplemented by herbarium specimens (27%). In total, all five sexual taxa and 37 apomictic taxa were represented by at least two populations (Table S1). Concerning the 64 morphologically assignable apomictic taxa, 30 taxa were represented by three or more populations. In general, three different leaf cycles are recognized within the *R. auricomus* complex [120,121,142,161] that roughly fit the three observed genomic RAD-Seq clusters (see [51] and Figure 4 in [120]). Taxa of cluster 1 usually have no dissected leaves at anthesis, which appear in clusters two and three on separate shoots (such additional shoots with dissected leaves can also be missing in stressed, small individuals of clusters 2 and 3, see [120,142,161,162], but such individuals were not included here). To avoid missing data for basal leaves (BL) of cluster 1, we took the functionally equivalent most-dissected summer leaves, which appear during early anthesis (of the next shoot; see [161]) for joint analyses with clusters 2 and 3, as in [39] (see also trait selection for GM analyses below). We additionally evaluated populations of genetic cluster 1 and genetic clusters 2 and 3 in some analyses separately (e.g., Figures 5 and 6).

#### 2.3.2. Digitalization of Traits and Extraction of Shape Variables

Image processing and the creation of TPS files followed the strategies described in [39,88]. The concatenated GM dataset of the sexual, di- to tetraploid populations was already published by [39] and added to the polyploid apomicts evaluated for the first time in this study. Herein, 2D landmark data of basal leaves (twenty-six landmarks), stem leaves (eight landmarks, twenty semilandmarks), and receptacles (nine landmarks, ten semilandmarks) were recorded using TpsDig version 1.4.0 [163]. The TPS-formatted raw datasets consisted of 4070 basal leaf configurations (BL), 4148 stem leaf configurations (SL), and 3472 receptacle configurations (RT). The three morphometric datasets were subjected to Procrustes superimpositions in TpsRelw version 1.70 [163] and MorphoJ version 1.07d [164] as described in [39] and only the symmetric component was further used to extract shape variables. Because most of the subsequent data analyses were based on population-level comparisons, the GM datasets were first averaged accordingly. Before the extraction of shape variables, landmark configurations were averaged across the same traits within each plant and across multiple plants within each population. Thus, for each population, we obtained symmetrized and averaged basal leaf, stem leaf, and receptacle configurations, each containing information from several plant individuals. Shape variables were calculated as scores of the symmetrized averaged landmark configurations (population means) on the shape principal components, also known as relative warps (RWs). In some analyses (e.g., in trait covariation analysis, PLS), the traits were analyzed separately, and in others, they were concatenated into a single morphometric dataset (e.g., in multi-group discriminant analyses).

#### 2.3.3. Genomic Clusters and Morphological Groups

We performed a multi-group discriminant analysis (Canonical Variates Analysis, CVA) of single-trait and concatenated GM datasets to investigate which genomic dataset best reflected the morphological differentiation. In the CVAs, we compared the morphometric distances between population clusters whose composition was inferred from analyses of genome-wide RAD-Seq data (three-cluster scenario), nuclear TEG (five-cluster scenario), and plastomes (four-cluster scenario). For these comparisons of morphological groupings, morphometric data for 66 populations were used, for which all three NGS datasets were available. Wherever the three traits were analyzed separately in the software MorphoJ, the Procrustes and Mahalanobis distances of the group centroids were calculated, including permutation tests of significance. In concatenated trait analyses using the software PAST version 4.11 [165], differences between group centroids were captured by Euclidean metrics and approved by permutation tests (NP-MANOVA, two-group permutation test). Results showed (see below) that RAD-Seq (RADpainter) clusters best explained the observed morphological differentiation in single-trait and concatenated GM analyses (Table S2). Consequently, we used the three genetic clusters inferred from RADpainter analysis as the grouping for subsequent GM analyses.

#### 2.3.4. Covariation of Traits, Taxonomic Resolution, and Shape Changes along Genomic Gradients within Clusters

The three traits (BL, SL, and RT) were examined for the independence of their shape variation. The basic question of whether, for example, the basal leaves vary completely independently of the stem leaves was analyzed. We tested whether there is a significant covariance structure between any two traits, employing a partial least squares (PLS) analysis in the software MorphoJ. Trait covariance analyses were performed separately for each of the three observed RADpainter clusters. The significance of the covariance was determined by permutation tests, and the corresponding morphological trends of the traits were visualized as wireframe graphs. To study the resolution of described *R. auricomus* taxa within observed morphometric clusters, we conducted a CVA of 24 agamospecies with three or more sampled populations. Within each RAD-Seq cluster, the three morphological traits were analyzed separately to investigate their ability to distinguish between agamospecies.

To study morphological shape changes along genomic gradients, we calculated regression models between observed morphotypes and genomic background based on the RAD-Seq similarity matrix concerning all 220 populations. For each polyploid population, the RADpainter method was first used to determine how the four diploid/sexual subgenomes (C, E, F, and N) were represented in its polyploid apomictic genome. The percentages of the four subgenomes were used as predictor variables in a regression analysis to model the associated shape change of basal leaves, stem leaves, and receptacles. In other words, the regression model predicted the appearance of the traits depending on their genetic background. The regression models in the software TpsRegr64 version 1.50 [163] can visualize changes in the traits along gradients of given variables. Goodall’s F-test statistic was applied to the regression model, and its significance was determined by permutation tests with 10,000 rounds.

#### 2.3.5. Shape-Environment and Shape-Genomics Association Models

To infer the sources of morphological shape variation (e.g., environment or genetics; [81,82,95,166]), we calculated distance matrix-based multiple regression models (MRM) using the R package ‘ecodist’ version 2.0.9 [167]. First, we ensured that environmental factors and shape principal component axes (relative warps) were non-autocorrelated among all traits and for single traits (r > 0.8, Figures S2–S6) using the R-package ‘corrplot’ version 0.92. We transformed shape principal ordination components among all traits and per single trait into distance matrices based on Euclidean distances. Second, we transformed non-autocorrelated environmental characteristics of populations among all factors and per single factor into distance matrices based on Euclidean distances. Third, we imported the raw RADpainter similarity matrix into R and transformed it into a distance matrix using Euclidean distances. The normal distribution of distance matrices was checked by applying the basic R functions ‘qqnorm’ and ‘qqplot’. In all cases, we inferred non-normally distributed data. Finally, we used 211 populations with exact overlapping shapes, environments, and genomic RAD-Seq (RADpainter) information (3 × 22,155 data entries). We calculated four linear MRMs based on scaled (unit variance) variables, 1000 permutations, and Spearman rank correlations due to non-normally distributed data. A general MRM using shape distances as response and environmental and genomic distances (and their interaction) as explanatory variables, and three more detailed MRMs using shape distances of BL, SL, and RT as response variables and all single environmental factors and genomic distances as explanatory variables.

Subsequently, the inferred significant environmental variables were used to model their effect on a basal leaf, stem leaf, and receptacle phenotypes. The regression models of the association between shapes and variable gradients were computed in the software TpsRegr64 version 1.50, the model fit was tested by permutation tests with 10,000 rounds.

#### 2.3.6. Ancestral Shape Reconstruction

The approach of Section 2.3.4 reconstructed a three-lobed to -dissected ancestral BL type for each genomic cluster (see Results). To verify this finding and to model in detail the ancestral basal leaf shape at the root of the European *R. auricomus* complex, we performed the squared-change parsimony analysis [168,169] for reconstructing the ancestral BL shape based on a phylogenomic tree (inferred from RAD-Seq data; for the phylogenomic methodology see [51]), using MorphoJ. The ancestral shape reconstruction utilized only individuals with exactly overlapping GM and RAD-Seq data, i.e., six samples of *R. cassubicifolius* (non-dissected leaf morphotype), two samples of *R. flabellifolius* (non- and slightly dissected leaf morphotypes), two samples of *R. marsicus* (dissected leaf morphotype), two samples of *R. envalirensis* (dissected leaf morphotype), and twelve samples of *R. notabilis* (dissected leaf morphotype). After computing the shape changes across all nodes in the phylogenomic tree, we summarized them using the evolutionary principal components analysis (EPCA) in MorphoJ, to extract the most important shape-shifts in the BL morphological evolution.

#### 2.3.7. Inferring Morphological and Genomic Differentiation in an Ecological Context, and Intermediary Versus Transgressive Hybrid Patterns

Due to their taxonomic importance and discriminative power, we explored the BL variation at three different levels (among clusters 1–3, among apomicts and sexuals within clusters, and hybrids and their genomic progenitors according to results in [51]) to infer intermediacy versus transgressive hybrid patterns. We employed a set of different analyses in MorphoJ: (1) principal components analysis (PCA) to explore the main shape trends in a common morphospace of different apomictic clusters or among apomicts and sexuals, (2) canonical variates analysis (CVA) and two-group discriminant analysis (DA) to test predefined groups for their morphological differentiation (and mean classification accuracy), and (3) partial least squares (PLS) analysis to put the phenotypic variation of the apomicts and/or their progenitors into the context of associated environmental factors. We selected four environmental covariates that exhibited the strongest association with BL variation, namely altitude, BIO3 (isothermality), BIO8 (mean temperature of the wettest quarter), and BIO18 (precipitation of the warmest quarter). The resulting PLS scatter plots showed the BL shape variation (PLS 1 ordination axis from shape data) against an ordination axis extracted from the environmental variables. The PLS analysis calculates the size of the covariation between the two linked datasets and provides a permutation *p*-value (10,000 rounds) for the significance of the covariance model. The PLS analysis identifies which of the original environmental variables shows the highest correlation with shape variation in a given PLS covariance model. With the methodology described above, we compared four apomictic nothotaxa recently approved as allopolyploids by [51] and their progenitors, but also apomicts of clusters 1–3, sexuals and apomicts within the clusters 1–3, and eight apomictic taxa within cluster 2 (taxon-rich and with known progenitors).

To corroborate ecological (dis)similarity among and within clusters of sexual and apomictic populations, we performed a new non-linear, machine learning-based ordination technique, known as UMAP (uniform manifold approximation and projection for dimension reduction; [170]), using PAST. Ecological similarities were computed for 212 populations based on eight non-correlated variables (altitude, solar radiation, BIO1, BIO3, BIO4, BIO8, BIO9, and BIO18) and the Manhattan similarity index.

## 3. Results

### 3.1. Morphological Clustering with Genomic Background (RAD-Seq)

**Figure 3:**
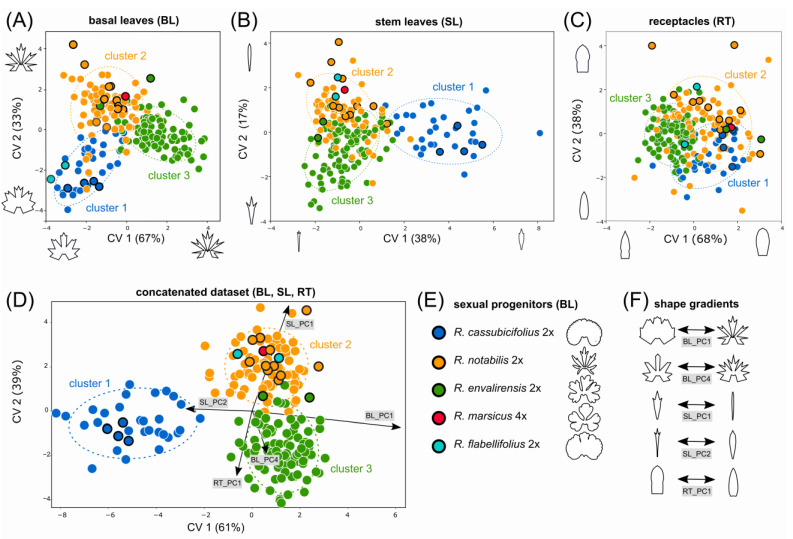
Morphological variation among sexual progenitors and polyploid apomictic derivative taxa with respect to genomic RAD-Seq (RADpainter) background. Canonical variate analyses (CVA) were applied for the clustering of 220 populations based on basal leaves (**A**), stem leaves (**B**), receptacles (**C**), and the concatenation of all traits (**D**). The concatenated analysis of all three traits (D) shows the five best separating morphometric trends illustrated in (**F**). Each dot in the CVA scatter plots (**A**–**D**) represents a single population, and the colors reflect assignments into the three RADpainter clusters (i.e., genomic clusters 1–3). An overview of the five sexual species in (**E**) shows their characteristic basal leaf morphotype, the most important taxonomic trait. The five best-separating shape trends (shape changes along the relative warps) are visualized in (**F**). The coloring of sexual progenitors and clusters follows Figure 2 and [51]. BL = basal leaves, Cluster 1 = RAD-Seq (RADpainter) cluster 1 containing *R. cassubicifolius* and polyploid apomictic relatives, Cluster 2 = RAD-Seq (RADpainter) cluster 2 containing *R. flabellifolius*, *R. marsicus*, and *R. notabilis* and polyploid apomictic relatives, Cluster 3 = RAD-Seq (RADpainter) cluster 3 containing *R. envalirensis* and polyploid apomictic relatives, CV = canonical variate (explained percentages of shape variation), SL = stem leaves, RT = receptacles.

Comparing the clustering of the 220 populations (Figure 3A–F; Figure S7) according to the three morphological traits, BL (Figure 3A) and SL (Figure 3B) exhibited similar patterns with a well-separated cluster 1 and partly separated clusters 2 and 3. The cluster separations are significant for each trait and the concatenated dataset, respectively (Table S2). The RTs (Figure 3C) exhibited the lowest discriminant power to distinguish genomic clusters 1 and 2, and 1 and 3 (Table S2). The concatenated dataset (BL + SL + RT) consisting of sixty-six shape variables separated genomic clusters best (Figure 3D). Almost all sexual progenitor species (Figure 3E) clustered consistently throughout the analyses, except for *R. flabellifolius* which clustered differently in the BL and SL analyses. Considering the concatenated analysis, out of the sixty-six input (shape) variables, we observed the following most important vectors (Figure 3D,F): The first (BL_PC1) and fourth (BL_PC4) basal leaf principal components, describing shape variation between non-dissected BL and narrowly, three- to five-lobed BL (cluster 1—cluster 2 and 3), and between narrowly, three-lobed BL with roundish segments and broadly, three-lobed BL with acuminate segments (cluster 2—cluster 3), respectively; the first two SL principal components (SL_PC1, SL_PC2), describing shape variation between broadly lanceolate SL with teeth and linear segments SL (cluster 1 and 3—cluster 2), and between linear SL with sinuses and oval segments SL (cluster 2 and 3—cluster 1), respectively; and the first RT principal component (RT_PC1), describing shape variation between broad and long androclinium and oval and short gynoclinium RT on the one side and short and narrow androclinium and high gynoclinium RT on the other side (cluster 2—cluster 1 and 3).

A detailed look at the morphospace trait occupation of the sexual progenitors and polyploid apomictic derivatives indicates the presence of transgressive apomictic phenotypes (compare Figure 3 and Figure S7). We detected a set of apomictic populations that grouped outside the range of sexual progenitors for all three traits and in the concatenated data analysis. Transgressive phenotypes were established along all three morphological traits and all three clusters but were most abundant in cluster 3.

### 3.2. Comparison of Morphological Clustering Concerning Different Genomic Backgrounds

**Figure 4:**
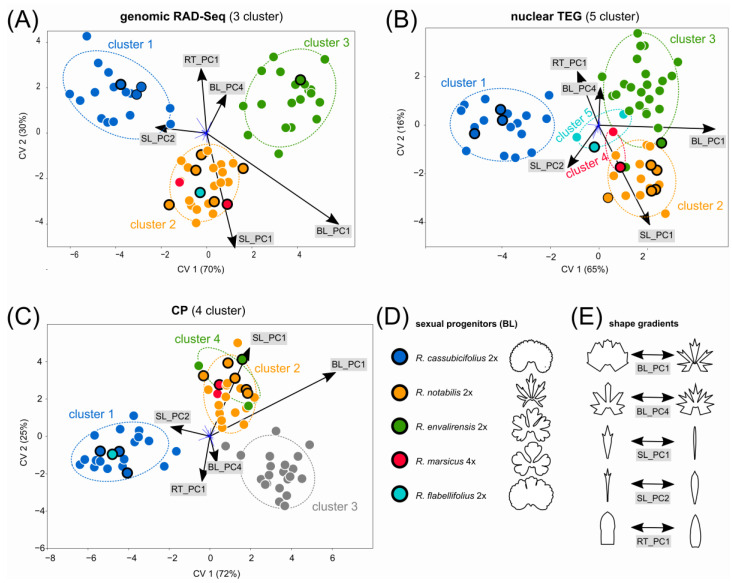
Morphological variation among sexual progenitors and polyploid apomictic derivative taxa with respect to different NGS backgrounds. Canonical variate analysis (CVA) was applied for the clustering of sixty-six populations based on the concatenated trait datasets (BL + SL + RT) and according to their assignment into clusters as inferred from genomic RAD-Seq (**A**), nuclear TEG (**B**), and CP (**C**) NGS-data (see also Materials and Methods for genomic cluster details). Each dot in the CVA scatter plots (**A**–**C**) represents a single population, and the colors reflect assignments into the RAD-Seq, TEG, and CP clusters (Table S1), respectively. Typical basal leaf morphotypes of the sexual species are shown in (**D**), and the best separating morphological trends are shown in (**E**). The coloring of sexual progenitors and clusters follows Figure 2 and [51]. BL = basal leaves; CP = plastid data; CV = canonical variate (explained percentages of shape variation); RAD-Seq = restriction-site associated DNA sequencing; RT = receptacles; SL = stem leaves; TEG = target enrichment nuclear genes.

We inferred significant morphological clustering according to genomic RAD-Seq (RADpainter, sNMF, NeighborNet clusters), nuclear gene TEG (Stacey clusters), and CP backgrounds (plastid clades) in CVA analyses (Table S2). Nevertheless, morphological clustering is best resolved by genomic RAD-Seq data showing the highest average differentiation value among clusters (F = 15.18 for RAD-Seq > F = 10.87 for CP > F = 8.29 for TEG; *p* values in Table S2; Figure 4A–E) inferred from the concatenated datasets. Though only representing a subset of sixty-six populations, the morphological groups correspond to those inferred from the analysis of 220 populations with RAD-Seq backgrounds (Figure 3D). The morphological clustering according to CP data was not able to distinguish *R. notabilis* (cluster 2) and *R. envalirensis* (cluster 4) and their respective polyploid apomicts from each other (Figure 4C). Concerning the TEG-guided clustering, morphological clusters 2–5 were highly overlapping, which was also indicated by the lowest average distance among the clusters (Table S2; Figure 4C). In general, *R. notabilis* and *R. cassubicifolius* clustered close to their polyploid apomictic relatives in RAD-Seq, TEG (Figure 4B), and CP (Figure 4C) analyses. In contrast, the morphological position of *R. envalirensis* and *R. flabellifolius* and their relationships to closely related polyploids were ambiguous throughout the analyses. The general morphological trends (Figure 4D), which best separated among the clusters, were identical to those inferred from the larger dataset of 220 populations (Figure 3, Table S3).

### 3.3. Covariation of Traits

**Figure 5:**
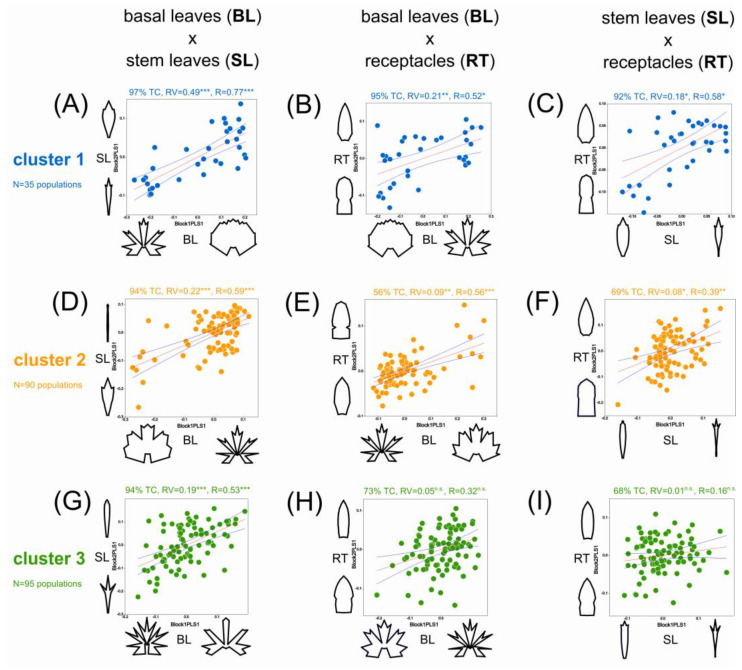
Covariation of the three taxonomically most informative traits is inferred for each RAD-Seq (RADpainter) cluster. The three morphological traits are plotted against each other in partial least-squares regression analyses of trait covariation. Within cluster 1 (inferred by RADpainter), the basal leaves are plotted against the stem leaves (**A**), against the receptacles (**B**), and the stem leaves against the receptacles (**C**). The same pairs of morphological traits were compared within clusters 2 (**D**–**F**) and 3 (**G**–**I**). Numbers above each plot give the amount of morphological covariation described by the first PLS axis (Block1PLS1 and Block2PLS1) as percentages of the total covariation (TC), a model fit statistic (RV) with its significance, and the correlation (R) of both PLS1 axes (each one representing one morphological trend). The coloring of sexual progenitors and clusters follows Figure 2 and [51], abbreviations as in Figure 3 and Figure 4. R = correlation coefficient of PLS axes; RV = global correlation coefficient (multivariate analog of the squared correlation); * = *p* < 0.05; ** = *p* < 0.01; *** = *p* < 0.001; TC = total covariance.

The covariation analyses revealed different trait behaviors among the three genomic RAD-Seq (RADpainter) clusters. The strongest significant association between basal leaves and stem leaves was found in cluster 1 (Figure 5A), showing a covariance between plants characterized by non-dissected BL with narrow blade base and broad lanceolate, teethed SLs and plants characterized by nearly five-dissected BL and narrow SL with sinuses. Within cluster 1, the relationships between the BL and RT (Figure 5B), and between the SL and RT (Figure 5C) shapes were much weaker, though significant. Within cluster 2, again BL and SL exhibited the strongest covariation structure (Figure 5D) compared to BL and RT (Figure 5E) and SL and RT (Figure 5F). Shape changes from broadly three-lobed BL with a narrow blade base and broad lanceolate teethed SL to narrowly, up to five-dissected BL with a broad blade base and sinuses and lineal SL segments. The covariation of BL and SL in cluster 3 (Figure 5G) was statistically similar to that described for cluster 2 but showed shape changes from broadly three- to five-dissected BL with a narrow blade base and deep sinuses and SL with deep sinuses to narrowly three-dissected BL with broad blade base. However, other covariation structures (BL and RT, SL and RT) within this cluster were non-significant (Figure 5H,I).

### 3.4. Morphological Clustering of Polyploid Apomictic Nothotaxa

**Figure 6:**
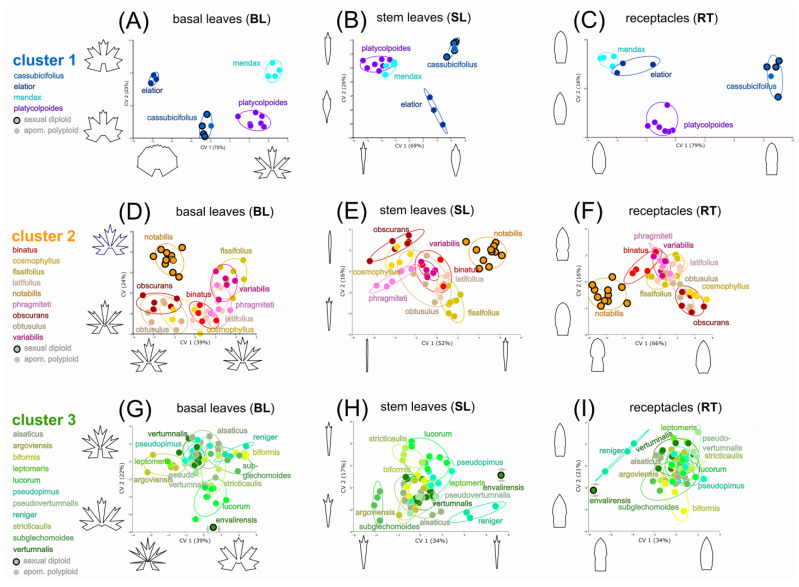
Morphological variation among sexual progenitors and polyploid apomictic derivative nothotaxa with respect to traits and genomic RAD-Seq (RADpainter) background. Canonical variate analyses (CVA) were applied for the clustering of twenty-four polyploid apomictic taxa based on the three morphological traits. Within cluster 1 (**A**–**C**), one sexual species and three apomictic polyploids were compared according to basal leaves (**A**), stem leaves (**B**), and receptacles (**C**). Within cluster 2 (**D**–**F**), one sexual species and eight apomictic polyploids were compared. In the case of cluster 3 (**G**–**I**), both sexual populations (*R. envalirensis*) were morphologically distant from all the polyploids, and their position within the plots was only graphically indicated by grey arrows. The coloring of sexual progenitors and clusters follows Figure 2 and [51]. BL = basal leaves, Cluster 1 = RAD-Seq (RADpainter) cluster 1 containing *R. cassubicifolius* and polyploid apomictic relatives, Cluster two = RAD-Seq (RADpainter) cluster 2 containing *R. flabellifolius*, *R. marsicus*, and *R. notabilis* and polyploid apomictic relatives, Cluster 3 = RAD-Seq (RADpainter) cluster 3 containing *R. envalirensis* and polyploid apomictic relatives; CV = canonical variate (explained percentages of shape variation); RT = receptacles; SL = stem leaves.

A detailed analysis of twenty-one polyploid apomictic nothotaxa and their respective sexual progenitor species pointed out similar patterns in all three genomic RAD-Seq (RADpainter) clusters. The sexual progenitors are clearly separated from the polyploid apomicts (nothotaxa) regarding all three morphological traits (BL, SL, RT; *p* values in Table S4). Within each RADpainter cluster, we observed that some polyploid nothotaxa were separated from each other across at least two different traits, but particularly in clusters 2 and 3, some polyploid nothotaxa strongly overlap in trait morphospace. We found a few examples of well-separated nothotaxa. For example, *R.* ×*platycolpoides* and *R.* ×*elatior* in cluster 1 (different in BL and SL, and partly in RT; Table S4), *R.* ×*fissifolius* and *R.* ×*obscurans* in cluster 2 (different in BL and SL, not in RT; Table S4), and *R.* ×*lucorum* and *R.* ×*reniger* in cluster 3 (different in BL and RT, not in SL; Table S4; Figure 6A–I). Nevertheless, the majority of polyploid apomictic nothotaxa overlap with another taxon across single or all traits. These are also often nothotaxa, which were hard to identify in the field, for example, *R.* ×*variabilis* and *R.* ×*phragmiteti* in cluster 2 (Figure 6D–F) or *R.* ×*alsaticus* and *R.* ×*vertumnalis* (Figure 6G–I) in Central Europe. In general, the weakest separation of nothotaxa was observed within cluster 3 (Figure 6G,H), showing highly overlapping trait variation.

### 3.5. Subgenome Contributions from Sexual Progenitors with Associated Morphotypes of Polyploid Apomicts, and Ancestral Morphotype Reconstruction

**Figure 7:**
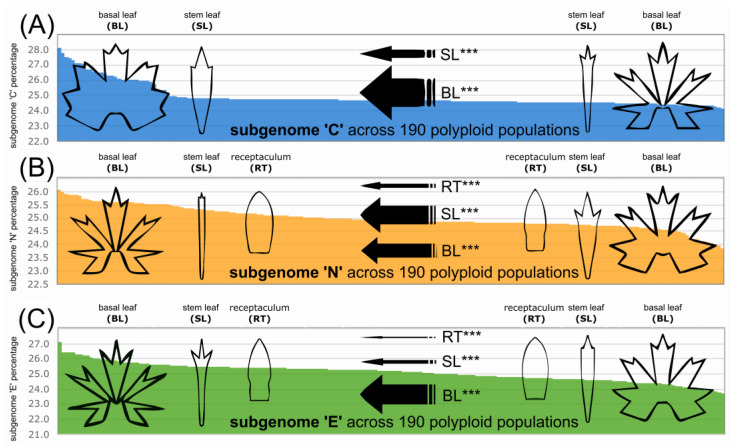
Morphological variation of polyploid apomicts along the gradients of subgenomic (RAD-Seq) contributions of sexual progenitor species. The multivariate multiple regression analyses are based on 190 polyploid apomictic populations. The polyploid morphotypes can be predicted by their genomic composition, namely by the sexual subgenomes predominating in the polyploid genomes. The regression models of shape in relation to genomic background were computed for the gradients of subgenome ‘C’ (**A**), ‘N’ (**B**), and ‘E’ (**C**). The illustrated morphotypes of basal leaves, stem leaves, and receptacles were generated by regression models, and all significant predictions are shown. The thicker the arrow, the stronger the association between trait shape and genomic composition. The coloring of sexual progenitors and clusters follows Figure 2 and [51]. BL = basal leaves; Cluster 1 = RAD-Seq (RADpainter) cluster 1 containing *R. cassubicifolius* and polyploid apomictic relatives; Cluster 2 = RAD-Seq (RADpainter) cluster 2 containing *R. flabellifolius*, *R. marsicus*, and *R. notabilis* and polyploid apomictic relatives; Cluster 3 = RAD-Seq (RADpainter) cluster 3 containing *R. envalirensis* and polyploid apomictic relatives; RT = receptacles; SL = stem leaves. *** = *p* < 0.001.

The genomic contributions of the three analyzed sexual progenitor subgenomes (*R. cassubicifolius* ‘C’, *R. notabilis* ‘N’, *R. envalirensis* ‘E’) were significantly associated with BL, SL, and RT shape variation across 190 populations of polyploid apomictic taxa. The increasing contributions of subgenome ‘C’ were associated most strongly with BL and less strongly with SL shape changes (*p* < 0.001; Table S5; Figure 7A). With increasing subgenome C contribution, the associated morphological shape converts towards less dissected BL phenotypes and broad lanceolate SL phenotypes (Figure 7A). An association between subgenome C contribution and RT shape variation could not be determined (Table S5). Variable contributions of subgenome ‘N’ were associated with BL (strongest association), SL, and RT shape variation (Figure 7B). With increasing subgenome N contribution, the BL shape of apomicts becomes more and more narrowly dissected, the SL shape becomes increasingly linear without sinuses, and the RT shape tends towards more roundish forms. Varying contribution of subgenome ‘E’ showed the strongest association with BL shape variation and a less pronounced association with SL and RT (Figure 7C; Table S5). With increasing subgenome E contribution, the BL shape of apomicts becomes more and more dissected with large sinuses, the SLs are narrower with sinuses, and the RT has a more roundish form with an elongated intervallum between the andro- and gynoclinium.

The reconstruction of the ancestral BL morphotype revealed an intermediary, three-lobed, and slightly dissected type at the root of the phylogenomic tree (Figure S8A), corroborated by the permutation test showing a significant phylogenetic signal in BL (*p* < 0.001). The evolutionary principal components analysis revealed that several shape changes (PC1-4) and not a single one (e.g., between non-dissected and dissected leaf types, as stressed out by several authors) played a role in the morphological evolution of the BL shape (Figure S8B). The two most important shape change components across the phylogenetic tree are concentrated at the incision and width of the middle segment and the blade base of a three-lobed to three-dissected BL (PC1/2; Figure S8B).

### 3.6. Environmental and Genomic Variables Associated with Phenotypic Variation

**Figure 8:**
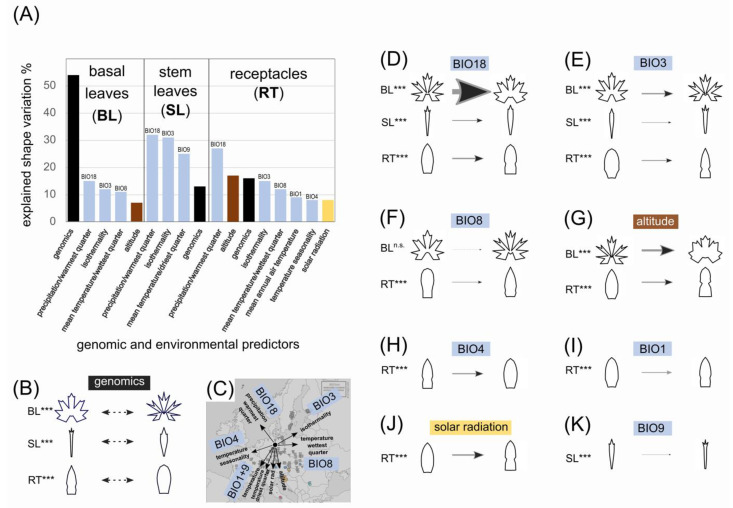
Shape variation of the three organs is associated with environmental variables and genomics. All variables with significant effects on shape variation are illustrated in (**A**), and the percentages give their relative importance for predicting the associated trait phenotypes. The genomic effects on shape (**B**) are visualized as shape variation at the first canonical variate, as shown in Figure 3. Environmental gradients affecting shape are plotted in the map of Europe (**C**) and the respective shape changes along the environmental variables were generated by regression models and visualized in (**D**–**K**). (**D**) The precipitation of the warmest quarter (BIO18) correlated to shape changes in BL, SL, and RT. (**E**) The isothermality (BIO3) is correlated to shape changes in B, SL, and RT. (**F**) The mean temperature of the wettest quarter (BIO8) correlated to shape changes in RT, (**G**) The altitude correlated to shape changes in basal leaves and receptacles. The RT shape changes were further correlated to the temperature seasonality (BIO4; **H**), the mean annual air temperature (BIO1; **I**), and solar radiation (**J**). The SL shape changes were also correlated to the mean temperature of the driest quarter (BIO9; **K**). The thicker the arrow, the stronger the association between trait shape variation and environmental predictors. *** = *p* < 0.001.

The calculated MRM revealed significant associations (*p* < 0.001, Table S6) between (i) overall shape distances (concatenated BL, SL, and RT datasets) among populations and their environmental distances (based on a set of eight environmental variables) and genomic distances (including their interaction), and (ii–iv) between morphological distances among populations (inferred from separate BL, SL, and RT datasets) and their environmental distances inferred from eight factors, and genomic distances. Overall, the shape distances among populations were strongly associated with their genomic distances (86%, *p* = 0.001), whereas environmental distance exhibited a relatively low explanatory power (14%, *p* = 0.011). When environmental and genomic distances were added to the same model, there was no change in the amount of explained variance. The BL shape distances among populations showed the strongest relationship with genomic distances (54%, *p* = 0.001) but also some associations with a couple of environmental factors (Figure 8A–C): precipitation of the warmest quarter (15%, *p* = 0.001; BIO18), isothermality (12%, *p* = 0.001; BIO3), mean temperature of the wettest quarter (11%, *p* = 0.01; BIO8), and altitude (7%, *p* = 0.033). In contrast to BL shape distances, which were associated mostly with genomic distances, SL and RT shape distances were more strongly associated with environmental factors than genomic distances (Figure 8A–C). SL shape distances exhibited associations with precipitation in the warmest quarter (32%, *p* = 0.001; BIO18), isothermality (31%, *p* = 0.001; BIO3), the mean temperature of the driest quarter (25%, *p* = 0.001; BIO9), and genomic distance (13%, *p* = 0.001). RT shape distances were associated with almost all environmental factors (precipitation in the warmest quarter > altitude > isothermality > mean temperature wettest quarter > mean temperature > temperature seasonality > solar radiation; in sum, 84%, *p* < 0.05) and genomic distances (16%, *p =* 0.002).

Regression models of shape variation (BL, SL, RT) in association with environmental variables (Table S7) showed a variety of morphological responses (Figure 8D–K). The precipitation of the warmest quarter (BIO18) was mostly associated with BL shape variation between finely dissected and more robustly dissected leaf phenotypes (Figure 8D). BIO18 has further associated with SL shape variation between finely dissected and more robustly dissected phenotypes (Figure 8D; Table S7) and RT shape variation between forms with a more pronounced intervallum and those without a distinct intervallum. The only other variable associated with the variation of all three traits (BL, SL, and RT) was isothermality (BIO3; BL > RT > SL; Figure 8E; Table S7). The BL shape variation correlated with BIO3 differs from that associated with BIO18, as the former varies not only with the fineness of the dissections but also with the blade base (Figure 8E). The SL shape variation correlated with BIO3 is opposite to that associated with BIO18, and RT varies between strongly pointed shapes and more rounded ones. The mean temperature of the wettest quarter (BIO8; Figure 8F) and altitude (Figure 8G) showed an association with shape variation in BL and RT, though the morphological trends differed between the two environmental predictors. The most pronounced morphological trend was the decreasing incision depth and blade base variation in BL associated with increasing altitude. The temperature seasonality (BIO4; Figure 8H), the mean annual air temperature (BIO1; Figure 8I), and solar radiation (Figure 8J) were correlated with the shape variation of RT (concerning mainly the appearance of the intervallum), with similar trends in BIO1 and solar radiation and an opposite trend in BIO4. The mean temperature of the driest quarter (BIO9) showed an association with SL variation between finer and more robustly dissected phenotypes (Figure 8K), similar to BIO3 (Figure 8E), and opposite to BIO18 (Figure 8D).

In a direct comparison of the apomicts from the tree clusters based on BL data, we observed morphological differentiation (Figure S9D–E). The jackknifed mean classification accuracy was 85% between clusters 1 and three and 79% for clusters 2 and 3. The morphological shift from more robust phenotypes of cluster 1 towards more gracile phenotypes of cluster 3 showed a significant covariation with four ecological variables, with the highest contribution being isothermality (BIO3; Figure S9F). Additionally, within the clusters, we observed differentiation between sexual and apomictic BL phenotypes (Figure S10). In cluster 1, the separation of sexuals and apomicts was the most pronounced (Figure S10A–C), while in cluster 2, it was the lowest among the three clusters (Figure S10D–F). In cluster 3, the differentiation among sexuals and apomicts was also significant (Figure S10G–I). The morphological differentiation exhibited covariances with ecological variables for cluster 1, we identified a shift towards drier climates by the apomicts (Figure S11A), while in clusters 2 and 3, most apomicts shifted towards more lowland climates than their sexual progenitors (Figure S11B,C). Among the apomictic taxa within the clusters, we could also identify morphological differentiation between more extreme BL morphotypes (e.g., *R.* ×*obscurans* and *R.* ×*binatus* in cluster 2; Figure S12A,B). The observed shift in BL phenotype showed a significant covariance with ecological variables (Figure S12C,D).

### 3.7. Morphologically and Ecologically Intermediate to Transgressive Polyploid Hybrids

**Figure 9:**
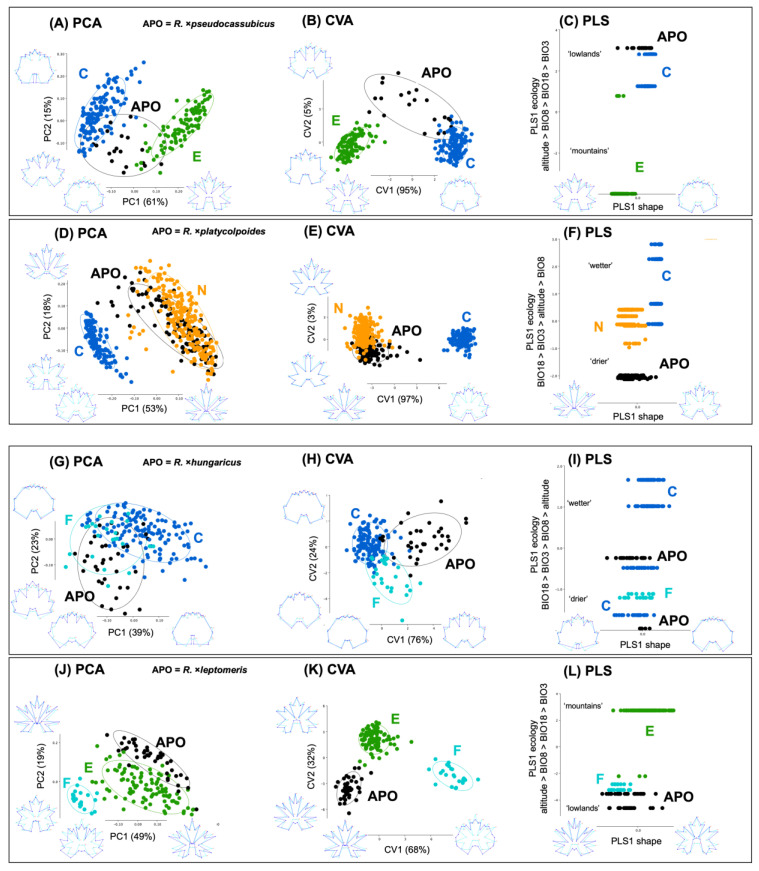
BL variation of sexual species and their selected allopolyploid apomictic (APO) derivatives. (**A**–**C**) *R.*×*pseudocassubicus*; (**D**–**F**) *R.* ×*platycolpoides*; (**G**–**J**) *R.* ×*hungaricus*; (**K**,**L**) *R.* ×*leptomeris*. PCA, principal component analyses, CVA, canonical variate analyses; PLS, partial least square analyses, (**A**) PCA of *R.* ×*pseudocassubicus* (black dots) and two sexual progenitor species *R. cassubicifolius* (blue dots) and *R. envalirensis* (green dots). (**B**) CVA of the taxa in (A). (**C**) PLS of the taxa in (A). The first PLS axis described 99.6% of the covariance between BL shapes and the four ecological variables, and the covariance model had *p* < 0.001. The first ordination axis from the shape data (PLS1 shape) was significantly correlated (0.90; *p* < 0.001) with the first ordination axis from the ecological data (PLS1 ecology). The highest contribution to the ecological component was provided by altitude (–0.79). (**D**) PCA of *R.* ×*platycolpoides* (black dots) and two sexual species, *R. cassubicifolius* (blue dots) and *R. notabilis* (orange dots). (**E**) CVA of the taxa in (D); (**F**) PLS of the taxa in (D). The first PLS axis described 97% of the covariance between BL shapes and the four ecological variables, and the covariance model had *p* < 0.001. The first ordination axis from the shape data (PLS1 shape) was significantly correlated (0.63; *p* < 0.001) with the first ordination axis from the ecological data (PLS1 ecology). The highest contribution to the ecological component was provided by the precipitation of the warmest quarter (BIO18; 0.75). (**G**) PCA of *R.* ×*hungaricus* (black dots) and two sexual species, *R. cassubicifolius* (blue dots) and *R. flabellifolius* (turquoise dots). (**H**) CVA of the taxa in (G). (**I**) PLS of the taxa in (G). The first PLS axis described 55% of the covariance between BL shapes and the four ecological variables, and the covariance model had *p* < 0.001. The first ordination axis from the shape data (PLS1 shape) was significantly correlated (0.45; *p* < 0.001) with the first ordination axis from the ecological data (PLS1 ecology). The highest contribution to the ecological component was provided by the precipitation of the warmest quarter (BIO18; 0.93) (**J**) PCA of *R.* ×*leptomeris* (black dots) and two sexual species, *R. cassubicifolius* (blue dots) and *R. flabellifolius* (turquoise dots). (**K**) CVA of the taxa in (J). (**L**) PLS of the taxa in (J). The first PLS-axis described 97% of the covariance between BL shapes and the four ecological variables, and the covariance model had *p* < 0.001. The first ordination axis from the shape data (PLS1 shape) was significantly correlated (0.64; *p* < 0.001) with the first ordination axis from the ecological data (PLS1 ecology). The highest contribution to the ecological component was provided by the altitude (0.78).

*R.* ×*pseudocassubicus* from cluster 1 is an obligate apomictic polyploid hybrid of *R. cassubicifolius* (cluster 1) and *R. envalirensis* (cluster 3), and it exhibited an intermediate position between the parental species within the BL morphospace (Figure 9A,B). This taxon showed an ecological shift towards lowland climatic conditions, which are outside the parental range but more distant from *R. envalirensis* and closer to *R. cassubicifolius* (Figure 9C). *R.* ×*platycolpoides* from cluster 1 is an obligate apomictic polyploid hybrid of *R. cassubicifolius* (cluster 1) and *R. notabilis* (cluster 2), exhibiting an intermediate (but closer to *R. notabilis*) morphological position between its parental species (Figure 9D,E), and showing a pronounced ecological shift towards a drier climate outside the parental niche preferences (Figure 9F). *R.* ×*hungaricus* from cluster 1 is an obligate apomictic polyploid hybrid of *R. cassubicifolius* (cluster 1) and *R. flabellifolius* (cluster 2) with mainly transgressive BL phenotypes (Figure 9G,H) and mainly intermediate ecological preferences located inside the parental species niche space (Figure 9I,J). *R.* ×*leptomeris* from cluster three is an obligate apomictic polyploid hybrid of *R. envalirensis* (cluster 3) and *R. flabellifolius* (cluster 2), with pronounced transgressive BL phenotypes (Figure 9K,L). *R.* ×*leptomeris* exhibited a slight ecological shift outside the range of its parental species toward a more lowland climate with less extreme temperature fluctuations (Figure 9M,N). Moreover, the UMAP analysis of ecological similarity among sexuals and apomicts inferred substantial ecological shifts of the apomicts far outside of the range of the progenitor species (Figure S13).

## 4. Discussion

This study gathered and evaluated what is currently the largest landmark-based, multi-trait GM dataset available for an intricate polyploid plant species complex, comprising more than 11,000 trait measurements from 220 diploid sexual to polyploid apomictic populations from 80 *R. auricomus* taxa. The GM dataset was tested for congruence with groupings derived from genomic datasets (genomic RAD-Seq, nuclear TEG, plastid CP), morphological distinctiveness, and morphological and ecological novelty of polyploid apomicts, but also for the discriminating power of different traits and NGS datasets. Additional information on ploidy and reproduction modes, as well as ecological data, was included following an integrative taxonomic approach, as recommended by several authors [36,44,45,50,51]. Consequently, we were able to analyze for the first time, objectively and in detail, the phenotypic diversity of the polyploid apomictic *R. auricomus* complex under a comprehensive (phylo)genomic background [24,51]. We showed that (1) the three previously defined genomic clusters representing five sexual species and 75 apomictic *R. auricomus* taxa correspond to morphological groupings based on both basal leaves and all traits together, and genomic RAD-Seq, as opposed to TEG and CP datasets, best fits the morphological resolution; (2) the apomictic taxa usually overlap within the trait morphospace except for those taxa at the morphospace edges; (3) trait-based phenotypes are highly shaped by genomic composition and to a lesser extent by environmental factors; and (4) allopolyploid apomictic taxa, compared to sexual progenitors, resemble a mosaic of ecological and morphological intermediaries to novel (transgressive) biotypes.

### 4.1. GM Methodology

Both main directions of morphometric approaches, i.e., traditional morphometry [64,171] as well as geometric morphometrics [87,88], have already been applied in the *R. auricomus* complex using the taxonomically most informative leaf and fruit characters. The use of traditional morphometry has enabled, for example, the quantitative evaluation of a few closely related sexual and polyploid apomictic taxa [64,171]. Traditional morphometry has a methodological advantage in that it can measure things such as the length and width of a leaf blade, its ratio, stem height, or length of the carpellophore or fruits. What traditional morphometry cannot capture, however, is more complex, detailed shape information and changes within a single objective statistical analysis, which can be interpreted in an anatomical, ecological, and evolutionary context [95,97,98,172]. In the case of *R. auricomus*, particularly the basal and stem leaves appear to have seemingly infinite variation across taxa, which challenges quantitative taxonomic treatments. GM uses modern digitalization and mathematical approaches to turn landmarks or outlines into quantitative variables, which can subsequently be analyzed with up-to-date multivariate statistics. Currently, the most useful contribution of GM for species delimitation is to test morphological hypotheses within an evolutionary theory-based framework and to provide a metric that makes biological trait variation among taxa measurable and comparable.

The first GM application for *R. auricomus* by [87] quantified basal leaf variation in two sexual diploid progenitor species and one natural apomictic hybrid. The authors compared their variation to that of artificially produced hybrids generated from the same progenitor species. This study demonstrated that even a relatively small dataset and landmark scheme (14 landmarks; in the present study we analyzed twenty-six landmarks) can reveal fundamental phenotypic shape changes in basal leaves, particularly in the highly variable dissected forms (e.g., *R. notabilis* and *R. variabilis*). The follow-up study by [88] expanded the landmark scheme to morphometrically analyze not only the variable dissected leaf shapes but also the undissected types (e.g., *R. cassubicifolius*; Figure 1B, Figure S1A). The new approach used 26 homologous landmarks to capture shape changes among the genetically and morphologically most distantly related sexual progenitor species and their crossings. Many artificially produced hybrids could be traced back to polyploid apomictic morphotypes found in nature, and thus GM further corroborated the idea about the hybrid origin of the *R. auricomus* complex. This study also showed that even an apparent continuum of forms can be decomposed into previously unknown (cryptic) morphological clusters [39], further extending the landmark approach to include two new traits, i.e., stem leaves (SL) and receptacles (RT). This phylogenomic-morphometric study on sexual progenitor species of the *R. auricomus* complex laid the foundation for the incorporation of GM into the taxonomy of this intricate plant group. For the first time, the significance of these three taxonomically most informative traits for separation among taxa could be quantified, which corroborated the final taxonomic treatment. The present study particularly makes progress in extending the multi-trait, landmark-based GM approach of [39] to polyploid apomicts and different genomic NGS datasets within an evolutionary framework, supported by the knowledge of previous studies (e.g., progenitor species circumscription, ploidy and reproduction modes, polyploid apomictic clusters, and genome evolution).

Our GM approaches are mainly aimed at unraveling complex-wide relationships in morphological trait variability. Therefore, we disregarded shape changes at the fine-grained level that could lead to more precise discrimination of apomictic taxa. Such additional traits concern the shape of early spring basal leaves, color of shoots, indumentum of basal leaves, shape of teeth at basal leaf blade margins, and indumentum of the receptacle. Although these fine-grained traits were often used by taxonomists to describe taxa (e.g., [121,128,142]), they are usually inconspicuous in the field, and some of these characters are available only at specific developmental stages (e.g., a reddish color on shoots appears in early spring during sprouting but disappears later) or are not stable in cultivation [161]. However, most fine-grained traits are related to phenotypic plasticity and do not discriminate genomic clusters or species (e.g., as shown for the indumentum of the receptacle in [39]).

### 4.2. Congruence of Genetic and Morphological Clustering, and Taxonomical Implications

The concatenated GM dataset revealed three significantly differentiated morphological groupings that are largely congruent with previously observed genomic clusters observed in [51] (Figure 3, Table S2). This finding is surprising because, in the light of field sampling and garden observations, no clear morphological groupings could be inferred due to seemingly endless phenotypic variation (Figure 1B–G). Each grouping comprises a single or few sexual progenitor species surrounded by polyploid apomictic derivatives. From the genomic perspective, this pattern is unique compared to other plant species complexes (e.g., [70,173]), where frequently several progenitors are found within clades or clusters or only polyploid descendants are observed. The origin of polyploid apomictic *Auricomi* is shaped by the hybridization of sexual progenitors, followed by early hybrid segregation, backcrossing to parents, polyploidization, and gene flow among apomicts due to facultative sexuality, leading to substantial genome evolution and consequently subgenome dominance [51,64,87,88]. The grouping of apomictic morphotypes around sexual progenitors probably represents the consequence of subgenome dominance and thus phenotypic trait expression rather similar to the dominant parent (Figure 7). Subgenome dominance is frequently observed in young allopolyploids [6,174]. However, effects on allopolyploid phenotypic trait expression based on exact subgenomic contributions have been so far less regarded in non-model plants, and hence, this study sheds new light on genomic-based changes in phenotypic and ecological features of naturally occurring allopolyploids (Figure 8, Figure 9 and Figure S13).

Although the current GM approach is labor- and cost-intensive (but see Section 4.4 on perspectives), it proved its value by unraveling and characterizing the morphological differentiation within a large part of the *R. auricomus* complex for the first time (Figure 3D). Cluster 1 (including progenitor *R. cassubicifolius*) is characterized by non-dissected to three-lobed BL with a narrow blade base, broadly lanceolate SL with teeth, and RT with short and narrow androclinium and high gynoclinium. Cluster 2 (including progenitors *R. flabellifolius* and *R. notabilis*) shows three-lobed to five-dissected BL with wide blade base and roundish leaf segments, SL with slightly to non-dissected, narrow to lineal segments, and RT with broad and long androclinium and oval and short gynoclinium. Finally, cluster 3 (including progenitor *R. envalirensis*) exhibits three-lobed to five-dissected BL with narrow blade base and strongly dissected leaf segments, SL with slightly to strongly dissected, narrow to broad segments, and RT with short and narrow androclinium and high gynoclinium.

In detail, the GM analysis of single and concatenated traits (BL, SL, RT) demonstrated that RAD-Seq genetic clusters show a quantifiable degree of morphological differentiation (Figure 3, Figure 4 and Figure S7; Table S2), and confirmed the usefulness of the GM approach in [39] also for a polyploid apomictic species complex. The RAD-Seq dataset is more effective in resolving morphological patterns because it provides magnitudes more information in coding and non-coding regions than the TEG single-copy genes applied here [51], and subgenome dominance seems to be more pronounced than maternal effects (CP data, Figure 4E). The last finding is supported by diploid crossing experiments, where F_2_ hybrids showed equal ratios of maternal, intermediate, and paternal phenotypes [88]. Morphological differentiation is corroborated by an overall classification accuracy of 85% for assigning populations into the three clusters (concatenated trait dataset). The stem leaves mainly separated clusters 1 and 2 (91%), clusters one and three (94%), and the basal leaves mainly distinguished clusters 1 and 3 (91%). On average, the concatenated data separated all pairs of clusters slightly better than single best-separating traits (Table S2). Interestingly, the separation between clusters was only slightly affected by the inclusion or exclusion of sexual populations (Tables S2 and S3, Figure S9C–E), supporting the stability of inferred morphological groupings. The overall multi-trait morphological differentiation was highest between clusters 1 and 3, and the lowest between clusters 2 and 3 (Table S2), which is in congruence with genomic clusters inferred previously [51]. Cluster 1 is most distinct from all other clusters because of its unique non-dissected BL and broad lanceolate SL, which are largely congruent with previous classifications of “*R. cassubicus*” or “*R. cassubicus* group” of several authors [127,142,175]. In contrast to expectations, we can morphologically characterize the previously only genetically recognized clusters 2 and 3, providing a basis for an informal grouping concept for the species complex [120]. However, these clusters do not match previous taxonomic treatments of “*R. auricomus*” and an intermediate “*R. fallax*” group, but rather follow a geographical, and longitudinal differentiation of genetic clusters, as outlined in [24,51].

Basal leaves bear the most discriminative power, followed by significant contributions from stem leaves and receptacles. The importance of BL shape variation has been stressed by previous taxonomists but requires a careful comparison of leaves within their leaf cycles. The distinction of cluster 1 is alongside important differences in stem leaves also due to a basal leaf cycle without dissected leaves that appear in most individuals of clusters 2 and 3 on separate, adventitious shoots during anthesis [120,121,142,161]. Our approach of using the functionally equivalent final leaves of cluster 1 (fully developed during anthesis) for comparison between clusters follows taxonomic practice. However, except for the general dissection of BLs, other distinguishing characters are also present in the complex to-discriminate clusters. For example, BLs of clusters 2 and 3 differ mostly by the angle of the blade base and segment dissection of the BL and differences in the width and dissection of SL segments, as described above (Figure 3 and Figure S14). The strong correlation between basal and stem leaf shape changes is expected from the shared developmental background of leaf organs. In Ranunculaceae, developmental studies [176] revealed that the ancestral leaf type has a trilobed to the ternate blade, from which either undivided leaves (by faster growth of blade than of the segmental meristems) or dissected leaves (by further secondary divisions of the segments) are independently derived. Our reconstruction of the ancestral BL shape of sexual species led to the same conclusion (Figure S8A), that the root of the Eurosiberian *Auricomi* possessed a three-lobed BL morphotype. The analysis also showed that the BL contained a phylogenetic signal. Interestingly, the shapeshift between non-dissected and dissected BL phenotypes (PC3) was not the transition dominating the morphological evolution of the species complex but rather two divergent traits from an ancestral intermediate (trilobed) leaf type (Figure S8B). Notably, the primary leaves of the BL cycle in clusters 2 and 3 are also often trilobed (e.g., Figure 1A, leaves 1–3) and might still reflect the ancestral shape, from which then the following leaves develop differentially. In general, the morphological clustering within the complex matches the background of a young, less than 1.0-Myr-old polyploid complex (sensu [177]) with a low degree of differentiation. The marked congruence of genetic and morphological patterns of allopolyploid clusters rather speaks for cluster criteria after [32] with regard to the entire *R. auricomus* complex, whereas only the sexual progenitors can be properly treated as species in the sense of evolutionary lineages and non-overlapping genetic/morphological clusters [39]. A modern ancestor-descendant lineage concept after [33] is hard to apply for the obligate to facultative apomictic allopolyploid nothotaxa due to multiple origins of the same morphotype (polyphyly, [51]), and even not at a cluster-wide scale, due to genetic and morphological instability of clusters in space and time caused by ongoing reticulate evolution.

### 4.3. Sexual Species and Apomictic Derivative Taxa in Relation to Morphospace and Ecology, and Taxonomic Implications

At a fine-grained morphological scale, and at first glance, clear morphological differentiation of polyploid apomictic taxa was not recognizable (Figure 6), especially when analyzing all available observations per trait (Figure S9B). Taxa of RAD-Seq clusters 1–3 usually overlap within single-trait morphospace (BL, SL, RT), except for sexual progenitor species and those polyploid apomictic taxa toward the morphospace edges (Figure 6 and Figure S10). Closer inspection also revealed some polyploid apomictic taxa that consistently formed well-separated morphological groupings in all three traits (Figure 6 and Figure S12). In cluster 1, e.g., *R*. ×*platycolpoides* and *R.* ×*elatior* are separated by all three traits. Nevertheless, one should keep in mind that several taxa from boreal Finland and Russia ([134], described under “*R. cassubicus”* and “*R. fallax”*) were not sampled here and that the entire variability of cluster 1 is not yet fully documented. In clusters 2 and 3, our more comprehensive sampling for Central Europe shows that apomictic taxa appear largely intermingled, with a few exceptions exhibiting pairwise morphological differentiation. For example, the apomictic taxa *R.* ×*obscurans* and *R.* ×*binatus* (cluster 2) are distinguishable from each other in all three morphological traits (mostly by SL and RT) as well as in the concatenated trait dataset (Figure S12A–C). However, most polyploid apomicts seem morphologically intermingled due to the broad variability of characters and mosaic-like character combinations as typical for hybrids (e.g., [5,102]). The shape of the BL can even vary among shoots of the same clone [161,171], which could be explained by differential gene expression of subgenomes [174] in these allopolyploids. The allopolyploid origin of apomicts from at least four distinct sexual progenitors has resulted in hundreds of local and regional morphotypes [51]. These morphotypes lack the homogenizing effect of regular (obligate) sexuality and hence cannot form coherent lineages and frequently do not form phenotypic clusters that could be recognized as species (sensu [32]). Within genomic clusters, the sexuals separate from the apomicts due to reproductive barriers via different ploidy levels [178], which speaks against concepts that simply sink apomicts into sexual species (e.g., as for sexual autotetraploids in *R. cassubicifolius*, [179]). Moreover, agamospecies concepts are largely inapplicable because facultative sexuality is still present, especially in Central Europe [24]. A classification as nothotaxa [51], despite methodological issues (allopolyploids here are hybrids of hybrids), appears to be a pragmatic solution to link existing names to a morphotype and its type location and to separate apomicts formally from sexual species [120].

Morphological shape changes within the European *R. auricomus* complex are mainly associated with the subgenomic composition of the allopolyploids and overall genomic differentiation, and less so with abiotic environmental conditions (Figure 6, Figure 7 and Figure 8). Results suggest a predominant heritable (epi)genetic control and a minor environmental regulation, particularly of BL features (e.g., [180,181,182]). BL leaf shape follows the pattern of an increased degree of basal leaf incisions under drier and hotter environments, but interestingly, under isothermal climatic conditions, the BL thus becomes more and more dissected towards temperature and precipitation stress conditions (same partly applies for SL, Figure 8A,D,E). These leaf traits probably reflect both climatic dependencies with a geographical west-east gradient (continentality) and altitude (Figure 8). Changes in BL leaf segments, margin incision, and the number of teeth usually influence leaf surface area, stomatal conductance, transpiration, and thus leaf energy balance and temperature, representing adaptation to water-limited and/or climatically variable environments [183,184]. Since shape differences are mainly attributed to genomic differences in the *R. auricomus* complex, these different leaf shapes probably represent selective advantages in their respective environments, for example, large non-dissected BL taxa along relatively water-rich but rather continental streamside habitats (e.g., *R. cassubicifolius*, *R.* ×*pseudocassubicus*), or strongly dissected BL taxa in less continental but rather dry anthropogenic meadows in Central-Eastern Europe (e.g., *R. notabilis*, *R.* ×*variabilis*). Interestingly, garden experiments with different levels of soil nutrients did not reveal changes in leaf shape, and different light treatments influenced only the size of plants and the number of leaves but not the shape of BL [161]. This indicates the predominant genomic fixation of BL features and thus supports the findings of this study. In addition, the shape of the receptacle has so far occasionally been utilized for descriptions of apomictic taxa (e.g., [121,137,142,185]), but not for the main groups. Contrary to expectation, results revealed that the RT shape separates clusters, specifically clusters 2 and 3 (Figure 3D and Figure 7B,C), but not the taxa within these clusters (Figure 6F,I). The RT is probably shaped by a mix of genomic and climatic factors.

Allopolyploid apomictic taxa, compared to sexual progenitors, resemble a mosaic of ecological and morphological intermediate to novel (transgressive) biotypes (Figure 9). Intermediate biotypes in sympatry and the same ecological niche as their progenitors could create an unpleasant “smear” between distinct sexual species, making their circumscription in practice difficult (e.g., [64]). However, intermediate morphotypes in allopatry or in different ecological niches can be recognized separately from sexual species. For instance, *R.* ×*pseudocassubicus* showed an ecological shift towards lowland climatic conditions, which are more distant to its Pyrenean mountain progenitor *R. envalirensis* and closer to sympatric *R. cassubicifolius* but outside of the parental range (Figure 9C). *R.* ×*platycolpoides* exhibited a pronounced ecological shift towards drier climates in southern Finland, which occurs not only allopatrically to the Central European parents, but also outside the parental ecological niche preferences (Figure 9F). Moreover, the UMAP analysis of ecological similarity among sexuals and apomicts inferred substantial ecological shifts of the apomicts far outside the range of the progenitor species (Figure S13). Ecological niche shifts can contribute significantly to the range expansions of allopolyploid apomicts compared to their progenitors (“geographical parthenogenesis”) [9,24,186]. We also observed allopolyploids with intermediate ecology but transgressive morphology, i.e., with characters outside the parental’s morphospace (e.g., *R.* ×*hungaricus* and *R.* ×*leptomeris).* In general, our results support a general hypothesis of genomic and phenotypic novelty in allopolyploids [3,7,55,56,57].

### 4.4. Perspectives of GM and Species Identification

Our results corroborated the most recent taxonomic treatments of the complex for Central Europe, to separate sexual progenitors as species and to treat the allopolyploid derivates as nothotaxa that can be grouped into three main informal clusters [39,51,120]. Identification of described or new taxa, however, is still a challenge because of the mosaic-like diversity of character combinations. Recent technological advances in automatically identifying plant species using machine learning (ML) can also be used in genetic modification (GM) and, by extension, in systematic biology. Similar to the landmark approach described in this study, automatic plant identification in the beginning also relied on manual feature extraction. From images of leaves or flowers, morphological features such as leaf shapes, leaf margins, leaf textures, flower shapes, or flower color were extracted [187]. The respective developed model refers to these features in the subsequent classification step. In recent years, so-called artificial neural networks (ANNs, a type of ML), and, more specifically, convolutional neural networks (CNNs), have made significant breakthroughs in automatic image classification [188]. They are already used in automatic plant identification [189,190] as well as in the extraction of plant features from herbaria [191,192]. The computer independently learns to recognize the structure of data, sometimes using up to millions of plant images.

Defining and setting landmarks as described in this study is labor-intensive, subjective, and a task for experts only. A species-specific machine learning approach to setting homologous landmarks automatically along the outline of a specific plant organ would be desirable. Attempts to combine GM and ML have been recently made in anthropology [193] and zoology [194,195,196]. However, similar to automatic plant identification, features extracted from ANNs [197] can be used for morphometric analysis of plant organs. It would be worth testing whether ANNs can similarly distinguish the different groups as unraveled in the GM approach applied herein. Structured image datasets as created in this study allow the visualization of self-learned features using, for example, Grad-CAM [198] to infer which leaf or receptacle region in the input image makes a large impact on species classification. Subsequently, it would be possible to investigate what the machine ‘sees’ across images and compare these results with the GM approach based on landmarks defined by experts. Such comparisons would make an important contribution to the explainability of features extracted by ML and provide important insights for species delimitation and final taxonomic decisions.

## 5. Conclusions

The polyploid apomictic *Ranunculus auricomus* complex exhibits enormous variability in morphological traits, which is often the case for predominantly hybridogenic species complexes and TCGs. After previous studies identified a structure of three genetic clusters within the *R. auricomus* complex, in the present study, we searched for morphological differentiation between clusters and apomictic nothotaxa. Morphological differentiation among the genetic clusters could indeed be detected by an extensive sampling of populations across Europe and using quantitative geometric morphometrics. The basal leaves as well as concatenated-traits data proved particularly useful for the morphological differentiation of the clusters. The hitherto confusing diversity of trait phenotypes and trait phenotype combinations thus received a basic structure on which future taxonomic treatments can build. However, most of the agamospecies described so far within these clusters could not be discriminated against. Moreover, it was also possible to figure out whether the genetic background alone or both the genetic background and the abiotic environment have a big effect on phenotypic diversity in the *R. auricomus* complex. We demonstrated that the hybridogenic phenotypic variability of polyploid apomicts is predominantly genetically determined, which means that the hybrid phenotypes are strongly shaped by parental subgenome contributions. Nevertheless, a couple of environmental parameters (e.g., temperature, precipitation, and temperature variability) could be identified, which influence phenotypic trait expression of leaves and receptacles. While most hybridogenic apomictic nothotaxa are morphologically within but ecologically outside the range of their progenitors, transgressive phenotypes have also evolved. ML techniques in combination with genomics and morphometrics promise new opportunities for future research on plant phenotypic variation and differentiation and integrative taxon-omics.

This study confirms a concept of classification proposed by [39], in which only sexual taxa represent well-defined species, while apomictic hybridogenic taxa are classified formally as nothotaxa [51,120]. The three big genetic and morphological clusters found here represent a geographical structure, but are not congruent with traditional taxonomic treatments of “main species” sensu [122,134]. These clusters will provide the foundation for a novel taxonomic treatment applying either a cluster criterion-based approach [38] or another infrageneric category, as soon as the whole complex has been analyzed. Our study highlights that detailed genomic and morphometric studies are needed to understand the evolution and structuring of agamic TCGs, which is required for a modern evolutionary classification. Traditional descriptive morphological treatments, however, failed to recognize taxa as natural entities on all levels of the hierarchy. Our study exemplifies a timely approach to the classification of TCGs.

## Data Availability

The authors declare that basic data supporting the findings are available within the manuscript and Supporting Information. RAD-Seq and target enrichment reads are deposited on the National Center for Biotechnology Information Sequence Read Archive (SRA): BioProject ID PRJNA627796, https://www.ncbi.nlm.nih.gov/bioproject/627796 (accessed on 7 March 2023) and BioProject ID PRJNA628081, https://www.ncbi.nlm.nih.gov/bioproject/628081 (accessed on 7 March 2023), respectively. More detailed data, tables, and figures concerning (phylo)genomic analyses are deposited on FigShare (https://doi.org/10.6084/m9.figshare.14046305) (accessed on 7 March 2023). Flow cytometric (FC) and flow cytometric seed screening (FCSS) data (ploidy levels, reproduction modes) are also stored in Figshare (https://doi.org/10.6084/m9.figshare.13352429) (accessed on 7 March 2023). We deposited image data processed for geometric morphometric analyses on FigShare upon publication (https://doi.org/10.6084/m9.figshare.21393375) (accessed on 31 January 2023).

## Supplementary Materials

The following supporting information can be downloaded at: https://www.mdpi.com/article/10.3390/biology12030418/s1, Figure S1: Landmark digitization of the taxonomically most informative *Ranunculus auricomus* traits; Figure S2: Correlation plot concerning non-autocorrelated (r < 0.8), standardized (0 mean, unit variance) abiotic environmental factors; Figure S3: Correlation plot concerning standardized axis shape scores of all taxonomically informative traits; Figure S4: Correlation plot concerning standardized axis shape scores of basal leaves; Figure S5: Correlation plot concerning standardized axis shape scores of stem leaves; Figure S6: Correlation plot concerning standardized axis shape scores of receptacles; Figure S7: Morphological variation among sexual progenitors and polyploid apomictic derivative taxa with respect to genomic RAD-Seq (RADpainter) background; Figure S8: Ancestral basal leaf (BL) shape reconstruction using the sexual progenitors; Figure S9: Basal leaf (BL) differentiation among clusters: apomictic polyploids; Figure S10: Basal leaf (BL) differentiation within clusters: sexuals vs. apomicts; Figure S11: Basal leaf (BL) differentiation within clusters: ecological covariance; Figure S12: Morphological differentiation among apomictic polyploids; Figure S13: Ecological clustering of sexual and apomictic populations; Figure S14: Selected sexuals and apomicts showing consistent genetic+morphological clusterings; Table S1: Information on 220 populations with complete evidence of collection (GPS), morphometric (BL + SL + RT), and genomic/RAD-Seq clustering data; Table S2: Discriminant analysis of the differentiation among genomic (RAD-Seq), nuclear (TEG), and plastid (CP) clusters based on pairwise comparisons of single morphological traits and concatenated data; Table S3: Discriminant analysis of the differentiation among the three genomic (RAD-Seq) clusters based on pairwise comparisons of single morphological traits and concatenated data; Table S4: Discriminant analysis of the differentiation among selected nothotaxa within each of the three genomic (RAD-Seq) clusters based on pairwise comparisons of the three morphological traits; Table S5: Regression models of the association between genomic contributions of the sexual species and shape variation in apomictic polyploids (mostly allopolyploids); Table S6: Distance matrix-based multiple regression models (MRM) to explain the source of shape variation; Table S7: Regression models of the association between eight non-correlated ecological predictors and variation of all three morphological traits.
